# Indirect pathway neurons in the tail of the striatum regulate inhibitory control over sensory driven behavior

**DOI:** 10.1126/sciadv.aeb5352

**Published:** 2026-07-31

**Authors:** Sarah M. Ferrigno, Evan Iliakis, Nathan Zhang, Saurabh Pandey, Jamie Galanaugh, Jonibek Muhsinov, Marc V. Fuccillo

**Affiliations:** ^1^ Department of Neuroscience, Perelman School of Medicine, University of Pennsylvania, Philadelphia, PA 19104, USA.; ^2^ Neuroscience Graduate Group, Perelman School of Medicine, University of Pennsylvania, Philadelphia, PA 19104, USA.; ^3^ Autism Spectrum Program of Excellence, University of Pennsylvania, Philadelphia, PA 19104, USA.; ^4^ Medical Scientist Training Program, University of Pennsylvania, Philadelphia, PA 19104, USA.

## Abstract

Inhibitory control, the ability to withhold action in certain contexts, is behaviorally essential. Disrupted inhibitory control is linked to various neuropsychiatric symptoms, making it critical to understand the underlying neural basis. We examined how the tail of the striatum (TS), a major basal ganglia sensory hub, regulates actions to sensory stimuli. Mice performed an auditory Go/NoGo task, while we recorded TS neuron activity. Both spiny neuron subtypes were recruited during target and nontarget sounds, but nontarget sounds uniquely engaged persistent indirect pathway neuron activity. Temporarily silencing this activity increased errors to nontarget stimuli, indicating a role in suppressing inappropriate action. In mice deficient for Neurexin1α, a gene linked to ASD and ADHD, TS indirect pathway recruitment was reduced, and these mice demonstrated auditory-specific inhibitory control deficits that were ameliorated by boosting indirect pathway excitability. These findings highlight a subcortical target to potentially improve attentional and behavioral regulation in neurodevelopmental disorders.

## Teaser

Posterior striatal circuits control sensory-guided actions and are disrupted in a rodent model of neurodevelopmental disorders.

## INTRODUCTION

Inhibitory control, or the ability to suppress inappropriate urges or actions depending on situational context, is an essential part of daily function. Disruptions to this process likely drive impulsivity and attention deficits found in many neurodevelopmental disorders (NDDs). Traditional models have typically framed inhibitory control in terms of two distinct mechanisms—reactive inhibitory control, thought to be driven by bottom-up responses to unexpected sensory input, and proactive control, guided by top-down restraint in alignment with current goals (*1*–*4*). However, these binary frameworks have failed to fully align with emerging evidence, and more recent proposals have conceptualized inhibitory control as a complex, continuous process shaped by the interaction of multiple mechanisms across broad neural networks (*5*–*7*). In line with this, the classical view of inhibitory control as being primarily mediated by frontal executive circuits has expanded to include the basal ganglia, whose established role in goal-directed behavior suggests that they could serve as critical subcortical contributors to these cognitive processes (*1*, *2*, *8*).

The basal ganglia are an evolutionarily conserved set of subcortical nuclei that can select and amplify cortical activity while simultaneously regulating midbrain targets (*9*, *10*). While early experiments focused on the role of basal ganglia stimulation in generating movement (*11*, *12*), recent work has revealed a broader involvement in regulating behavior. As the primary input structure of the basal ganglia, the striatum receives topographically organized projections from the entire neocortex, making it well suited to integrate goal-directed strategies and sensory context with action selection (*13*, *14*). Cortical signals are processed in the striatum primarily through recruitment of its main neuronal population, the spiny projection neurons (SPNs) (*15*). These neurons comprise roughly 95% of striatal neurons and consist of two equally abundant subtypes with opposing effects on basal ganglia output (*16*–*18*). Dopamine D1 receptor-expressing direct pathway SPNs (dSPNs) and D2 receptor-expressing indirect pathway SPNs (iSPNs) are thought to promote respective excitation and inhibition of downstream thalamic neurons through distinct projections to intermediary basal ganglia nuclei (*18*).

Given that iSPNs suppress thalamic output in part through polysynaptic disinhibition of the subthalamic nucleus, a region previously implicated with inhibitory control, the indirect pathway is likely a key circuit element regulating control over inappropriate actions (*1*, *2*). iSPNs have been implicated in promoting a range of inhibitory motor and nonmotor avoidance behaviors (*19*–*23*). In addition to its downstream connectivity, the role of iSPN-mediated lateral inhibition within striatum has gained increased attention (*21*, *24*–*26*). Recent work has revealed that optogenetic activation of iSPNs in the dorsomedial striatum (DMS) induces motor slowing through a pathway distinct from the downstream pallidal effects on transient punishment (*21*). Together, this suggests that iSPNs can regulate inhibitory control over actions through multiple means, making them a compelling candidate for further investigation.

As the striatum receives widespread input, understanding how iSPNs contribute to sensory associated inhibitory control requires identification of the relevant striatal subregions integrating sensory input with higher-order goals. While there is an established literature in perceptual decision-making within anterior striatal circuits (*27*–*31*), recent work has identified the posterior tail of the striatum (TS) as an additional multimodal sensory integration domain (*32*–*36*). In line with this, several studies have shown that pathway-specific manipulation of SPNs within the TS is sufficient to bias lateral auditory choices in two-alternative forced-choice behavioral tasks (*26*, *37*, *38*). In addition to auditory processing, the TS has also been implicated in modulation of avoidance responses to threatening stimuli, and recent work has identified contributions of TS dSPNs and iSPNs for promoting or overcoming threat avoidance, respectively (*39*). Together, it seems possible that the iSPNs in the TS serve as mediators of inhibitory control across different situational contexts. Findings in rhesus monkeys show that the globus pallidus externa is involved in the rejection of low-value visual targets (*40*, *41*). However, to date, TS iSPN involvement in sensory-driven inhibitory control has not been investigated at a cellular level.

While a growing body of work has implicated striatal synaptic dysregulation in genetic models of NDDs (*17*, *42*–*45*), the specific contributions to disease-relevant behavioral changes remain understudied. Specifically, the potential role of iSPN dysfunction in mediating the impulsivity and attentional perturbations of NDDs is unknown. Recent work in slice has shown that mice lacking either one or both Neurexin1α (Nrxn1α) alleles exhibit synaptic deficits for PFC connections onto iSPNs in the DMS (*46*). Neurexins are presynaptic cell-adhesion molecules that regulate the development and maintenance of synapses throughout the brain (*47*–*49*). Copy number variations affecting the Nrxn1α isoform have been associated with increased risk for schizophrenia, autism spectrum disorder (ASD), attention deficit hyperactivity disorder (ADHD), and Tourette’s syndrome (*50*–*57*). Furthermore, Nrxn1α knockout (KO) mice likely have impaired sensory-related inhibitory control, manifest as reduced prepulse inhibition (*58*). However, whether these striatal circuit dysfunctions contribute to sensorimotor impulsivity seen in NDDs remains to be directly assessed.

To investigate the underlying circuits and molecular mediators of inhibitory control, we designed a lick-based Go/NoGo task where lick responses to distinct bandlimited sounds were either rewarded with water or punished with a time-out. We recorded pathway-specific SPN recruitment via fiber photometry as mice performed this auditory-guided task. While early neural responses to sound were not different between Go and NoGo trials in either SPN subtype, we uncovered a persistent NoGo-associated activity in iSPNs that our encoding model attributed to lick withholding. Consistent with a putative role in suppressing responses to inappropriate auditory stimuli, we found that the optogenetic inhibition of iSPNs but not dSPNs is sufficient to increase erroneous false alarm responses. We also observed decreased excitatory synaptic recruitment of TS iSPNs in Nrxn1α heterozygotes, revealing a disruption in TS iSPN circuits potentially relevant for associated NDDs. To assess whether this circuit dysfunction also accompanied impaired inhibitory control over sound driven behavior, we trained Nrxn1α KO mice in our task and found that they exhibited specific increases in false alarm errors that could not be explained by general motor impulsivity. Furthermore, chemogenetically boosting the excitability of TS iSPNs was sufficient to suppress increased false alarm rates in Nrxn1α KO. Together, these data suggest that iSPN activity in the TS supports inhibitory control over auditory-driven actions. As Nrxn1α mutations have been linked with various NDDs, our findings provide important insight into a subcortical circuit for inhibitory control that may provide therapeutic access for attentional and behavioral dysregulation.

## RESULTS

**Fig. 1.**
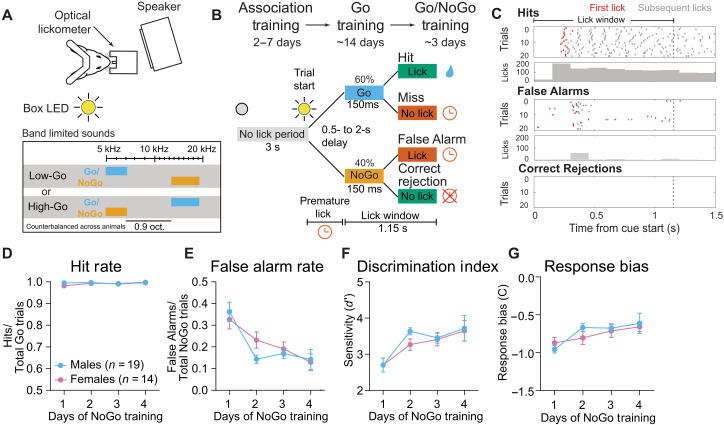
An auditory Go/NoGo task to study sensory-driven inhibitory control. (**A**) Behavioral apparatus and band-limited auditory stimuli. High- and low-frequency band-limited sounds were counterbalanced across animals as Go and NoGo cues. Illustration adapted from SciDraw under CC-BY 4.0 license (https://creativecommons.org/licenses/by/4.0/; mouse head schema, L. Petrucco, DOI: 10.5281/zenodo.3925903). (**B**) Training timeline and trial structure. (**C**) Lick rasters from an expert mouse showing Hit (top), False Alarm (middle), and Correct Rejection (bottom) trials aligned to cue onset. Each row represents one trial. First licks are shown in red, and subsequent licks are in gray. The gray dotted line indicates the end of the response window. Histograms (150-ms bins) below the Hit and False Alarm rasters show lick distributions across trials. (**D** to **G**) Behavioral performance across the first 4 days of Go/NoGo training for male (*n* = 19) and female (n = 14) mice (means ± SEM). Linear mixed-effects models included training day (within-subjects) and sex (between-subjects) as fixed effects and subject as a random effect (Geisser-Greenhouse corrected). (D) Hit rate: no effect of training day (*F*_2.31,53.91_ = 0.66, *P* = 0.54) or sex (*F*_1,31_ = 1.23, *P* = 0.28). (E) False alarm rate: main effect of training day (*F*_2.17,50.59_ = 14.31, *P* < 0.0001); no effect of sex (*F*_1,31_ = 0.53, *P* = 0.47). (F) *d*′: main effect of training day (*F*_2.27,52.87_ = 14.84, *P* < 0.0001); no effect of sex (*F*_1,31_ = 0.76, *P* = 0.39). (G) Response bias: main effect of training day (*F*_2.75,64.04_ = 5.92, *P* = 0.0017); no effect of sex (*F*_1,31_ = 0.28, *P* = 0.60).

To investigate mechanisms of inhibitory control, we used an auditory Go/NoGo task in which mice were trained to appropriately respond or withhold responding to sounds associated with distinct outcomes (Fig. 1, A and B). Mice were head-fixed in a custom-made behavioral apparatus where lick responses were recorded by an infrared optical lickometer at the reward spout. A speaker delivered bandlimited auditory stimuli, which were assigned as either “Go” or “NoGo” sounds. During Go trials, if mice licked within 1.15 s of the target sound onset, then the trial was considered a “hit,” and animals received a 4-μl water reward. Conversely, if animals failed to register a lick response within the response window following Go sound presentation, then that trial was classified as a “miss,” and the mice received a 5-s lockout penalty. For NoGo trials, if an animal followed the nontarget sound with a lick response, then that trial was classified as a “false alarm,” resulting in a 5-s lockout penalty. However, if the mouse correctly withheld responses after the NoGo sound, then the trial was a “correct rejection,” which was unrewarded but bypassed the lockout. We used established signal detection theory metrics, including the discrimination index (*d*′) to quantify the ability to distinguish target and nontarget stimuli, calculated as the difference of the z-transformed hit and false alarm rates, and the response bias (*c*) to measure the subject’s overall tendency to favor one response over the other, independent of the sound properties (calculated as the negative average of the z-transformed hit rate and false alarm rate).

Mice were initially trained in trials containing only the rewarded Go sound, with the final stage of training introducing a random subset (40% of total trials) of nonrewarded NoGo sound trials requiring sound-related inhibitory control (Fig. 1B). Most animals could achieve high discriminative performance (*d*′ > 2) within 4 days of training, driven by a stable hit rate and declining proportion of false alarms (Fig. 1, D to F). While animals generally exhibited a bias toward licking, as indicated by negative response biases, these values became less negative with continued training (Fig. 1G). As no significant sex differences were observed in learning or performance of our Go/NoGo behavior, data from both sexes were pooled together for all subsequent experiments (Fig. 1, D to G, and fig. S1). Performance and acquisition trajectories did not differ on the basis of frequency assignments of the Go and NoGo cues (fig. S2, A to D).

### Global inhibition of TS selectively impairs lick responses to target stimuli

**Fig. 2.**
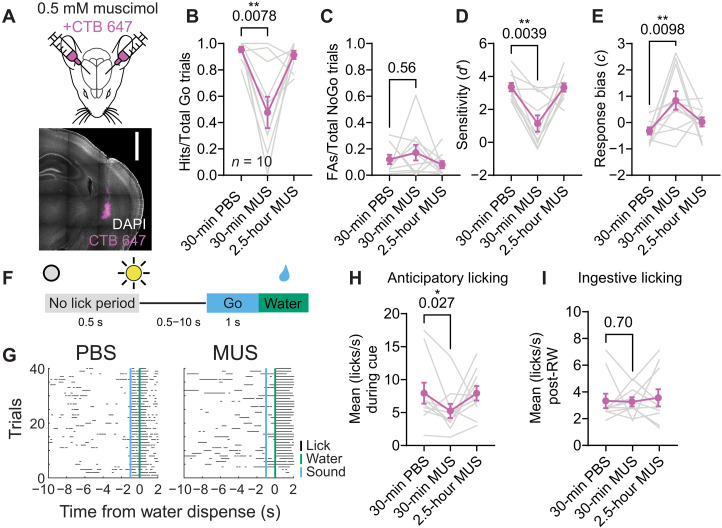
TS is required for appropriate target sound responses. (**A**) Experimental schematic for bilateral vehicle/drug administration with example histology showing the target injection site, visualized with cholera toxin subunit B, Alexa Fluor 647 conjugate (CTB 647; scale bar, 1 mm). High- and low-frequency sounds were counterbalanced as Go and NoGo cues across animals. Illustration adapted from SciDraw under CC-BY 4.0 license (https://creativecommons.org/licenses/by/4.0/; mouse head schema, L. Petrucco, DOI: 10.5281/zenodo.3925903). (**B** to **E**) Behavioral performance measured 30 min after PBS, 30 min after muscimol (MUS), and 2.5 hours after muscimol (*n* = 10; means ± SEM; gray lines connect individual mice). (B) Hit rate decreased following muscimol (Wilcoxon matched-pairs signed-rank test, 30-min muscimol versus PBS: *W* = −43, *P* = 0.0078). (C) False alarm rate was unchanged (*W* = 13, *P* = 0.56). (D) Discrimination index decreased (*W* = −53, *P* = 0.0039). (E) Response bias increased (*W* = 56, *P* = 0.0098). (**F**) Schematic of the non-operant session preceding each Go/NoGo session. (**G**) Example lick rasters 30 min after PBS (left) and muscimol (right). Licks are shown in black, sound onset in blue, and water delivery in green. (**H** and **I**) Lick rates during the non-operant session across conditions (*n* = 10; means ± SEM). (H) Lick rate during Go sound presentation was reduced following muscimol (*W* = −43, *P* = 0.027). (I) Lick rate after water delivery (RW) was unchanged (*W* = 9, *P* = 0.70). DAPI, 4′,6-diamidino-2-phenylindole.

To causally probe the contributions of TS to behavioral performance, we first used muscimol to broadly inhibit the region (Fig. 2). When animals reached “expert” performance on the Go/NoGo task (*d*′ > 2 on at least three consecutive Go/NoGo sessions), we bilaterally injected the GABA_A_ receptor agonist muscimol into the TS during expert Go/NoGo sessions, 1 day after administering phosphate-buffered saline (PBS) vehicle injections as a paired control (Fig. 2A). Muscimol administration significantly and reversibly suppressed responses to the target sound, while false alarm response rate remained unchanged (Fig. 2, B and C). This impairment in reward-related responding significantly reduced discriminative performance and resulted in a shift in an overall response bias toward withholding (Fig. 2, D and E). The effects of muscimol administration on Go/NoGo behavior had largely disappeared 2.5 hours following initial injection (Fig. 2, B to E).

To rule out motivational deficits or impairments in overall lick ability, before each Go/NoGo session, we also ran animals in a nonoperant version of the behavior where licking had no influence on reward delivery, and instead, reward was dispensed following a variable duration after trial start (Fig. 2F). This random reward delivery was consistently preceded by the presentation of the Go sound. During PBS sessions, animals predictably licked during the Go stimulus in anticipation of reward (Fig. 2, G and H). While muscimol administration significantly reduced this anticipatory licking, the ingestive lick rate after reward dispensing was not significantly altered (Fig. 2, G to I). This indicates that although animals exhibited an intact motivational drive for water reward and overall ability to lick to ingest reward, licking in response to the target stimulus was specifically disrupted.

### Indirect pathway SPNs track response withholding during NoGo trials

**Fig. 3.**
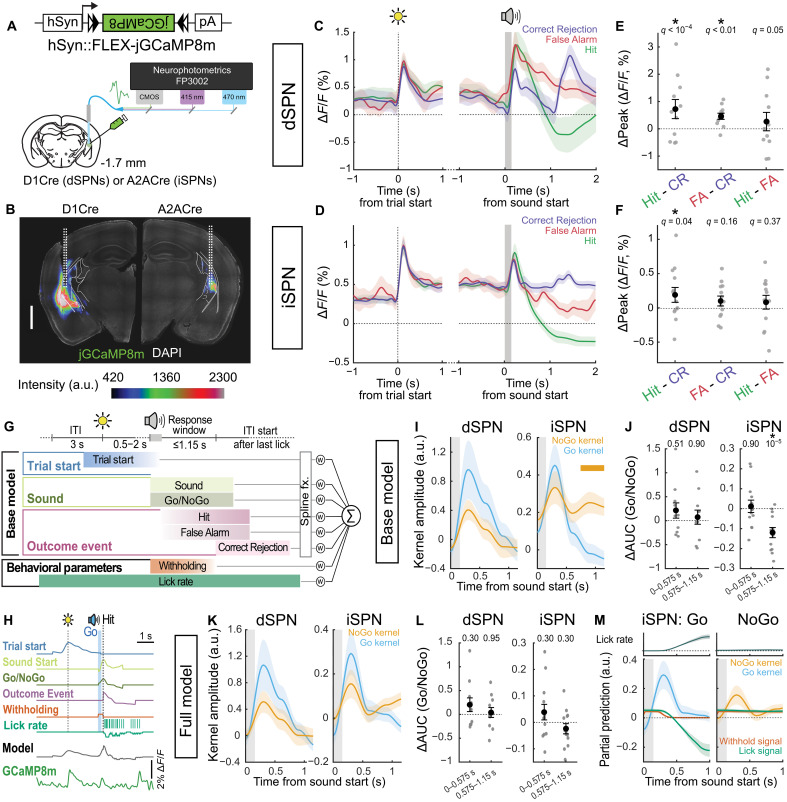
Enhanced recruitment of indirect pathway SPNs during response inhibition. (**A**) jGCaMP8m expressed in direct (dSPNs) or indirect (iSPNs) pathway neurons in the striatal tail. Atlas diagrams used with permission of Elsevier Science & Technology Journals, from “*The Mouse Brain in Stereotaxic Coordinates*” by G. Paxinos and K. B.J. Franklin, second edition, 2001 (permission conveyed through Copyright Clearance Center Inc). (**B**) jGCaMP8m expression in dSPNs and iSPNs (scale bar, 1 mm). (**C** and **D**) Mean Δ*F*/*F* aligned to trial start and sound onset for dSPNs (C; *N* = 11 animals, 20 sites) and iSPNs (D; *N* = 13 animals, 25 sites) by outcome. Shaded regions indicate animal-level SEM. Miss trials omitted due to low counts. Sound mapping counterbalanced across animals. Data from expert sessions (*d*′ ≥ 2 across three consecutive sessions). (**E** and **F**) Evoked peak responses for dSPNs (E) and iSPNs (F), per-trial as mean of five largest Δ*F*/*F* samples within 400-ms post-sound minus 500-ms presound baseline. Trial-type effects assessed using linear mixed-effects models [Peak ∼ TrialType + (1|Animal_ID)]; significant effects observed for dSPNs (*P* < 0.0001) and iSPNs (*P* = 0.041), with FDR-corrected pairwise comparisons (*q* < 0.05). (**G** to **M**) Kernel-based linear model separating sensory and behavioral contributions. (G) Base Model included event-aligned kernels; full model additionally included lick rate and response withholding regressors. Parameters estimated using ridge regression with fivefold cross-validation. (H) iSPN trial showing model prediction and recorded signal. (I) Sound kernels (base model) for dSPNs and iSPNs, separated by Go and NoGo; shaded region indicates significant iSPN differences (cluster-based permutation test). (J) Within-animal ΔAUC (Go/NoGo) for early (0 to 0.575 s) and late (0.575 to 1.15 s) windows analyzed using linear mixed-effects models with FDR correction. (K to L) Full model kernels and ΔAUC showing loss of late iSPN Go/NoGo differences. (M) Partial model predictions illustrating contributions of lick rate, response withholding, and sound kernels. a.u., arbitrary unit.

To observe SPN subtype recruitment as mice performed our auditory-guided task, we used fiber photometry to visualize direct and indirect pathway population activity. We selectively expressed the fluorescent calcium (Ca^2+^) sensor, GCAMP8m, in either D1- or D2-dopamine receptor-positive SPNs (dSPNs and iSPNs) by injecting a Cre-dependent adeno-associated virus (AAV) construct (AAV-hSyn::FLEX-jGCaMP8m) into the TS of D1Cre or A2ACre mice (Fig. 3, A and B, and fig. S4A). We did not observe any differences in behavioral performance for either of these transgenic strains (fig. S3, A to D). We first measured peak photometry responses to Go and NoGo sounds in our task design, in which both high- and low-frequency band-limited sounds served as Go or NoGo instructions across animals (Fig. 1A). Initial exposure to high-frequency tones reliably evoked larger peak responses than lower frequency tones, independent of task instruction and sound intensity (all frequencies presented at 65 dB), suggesting a potential degree of tonotopic bias detectable with TS photometry or greater sensitivity to high-frequency sounds (fig. S4, B and C). To control for this potential confound, sound-instruction mappings were counterbalanced across experiments: Half of the mice were trained with High-Go/Low-NoGo mapping, whereas the remaining animals were trained with Low-Go/High-NoGo mapping. Photometry data broken down by counterbalancing condition are shown in fig. S4 (D to M).

To obtain a broad overview of neuronal recruitment during expert performance (defined as any session following three consecutive sessions with *d*′ > 2), we aligned all raw photometry traces to trial start and sound onset, sorting them based on trial type (Fig. 3, C and D). We first observed that both SPN subtypes exhibited similar phasic responses to the light-associated trial-start cue, followed by a modestly elevated fluorescence that lasted until sound presentation (Fig. 3, C and D). In contrast, the sound-evoked Ca^2+^ waveforms markedly diverged according to SPN subtype. We first quantified the difference in peak fluorescence for all combinations of hit, correct rejection, and false alarm trials. For dSPNs, we noted that both hit and false alarm peaks were of similar magnitude and were both consistently larger than correct rejections (Fig. 3E). In contrast, for iSPNs, we only noted a small positive difference between hit and correct rejection trials (Fig. 3F). Miss trials were omitted as their low frequency made it challenging to estimate average activity. Notably, we observed phasic increases in dSPN and iSPN signal on correct rejection trials, coinciding with the end of the response window, 1.15 s after sound start (Fig. 3, C and D).

We also noted complex trial-type and SPN subtype dynamics following the sound presentation that likely represent an interplay between neural activities associated with licking, lick withholding and a range of trial-specific outcomes. To better resolve these partially overlapping signals, we modeled SPN Ca^2+^ signals as a linear sum of task-relevant regression parameters. In our “base” encoding model, we included spline-based, temporally expanded kernels aligned to light onset (“trial start”), sound presentation (separate kernels for the “sound” event per se—agnostic to instruction and frequency—and “Go/NoGo instruction”), and trial outcome (Fig. 3G). Leveraging the temporal variability in our behavior, we fit these regression parameters with a generalized linear model (details in Materials and Methods) to estimate kernels that roughly capture the Ca^2+^ signal components associated with temporally separated, behavioral events (Fig. 3, G and H, and fig. S5A).

Because our model simultaneously accounted for trial start, sound, and outcome events, the estimated kernels reflect sound-evoked fluorescence changes after variance attributable to other task events has been absorbed by their respective regressors. We therefore focused on neural Ca^2+^ signals beginning at sound presentation, when auditory information must be used to adapt motor output. We first investigated the Go/NoGo sound kernels, quantifying them by measuring the difference in area under the curve (ΔAUC) between Go and NoGo trials per animal. Given the 1.15-s response window and the observed kernel structure, we split this analysis into early (0 to 0.575 s) and late (0.575 to 1.15 s) post-sound periods. In the early window, the ΔAUC did not differ between Go and NoGo trials for either SPN subtype (Fig. 3, I and J). In the late window, dSPNs showed similar activity patterns across trial types, whereas iSPNs exhibited a markedly larger sustained signal on NoGo trials (Fig. 3, I and J). This late divergence in iSPN signal following the sound presentation suggests the presence of additional behaviorally relevant predictors not captured in our base encoding model.

To address this, we expanded the encoding model to include two motor-related parameters: lick rate and an active response-withholding process (Fig. 3, G and H). We tested these additions both independently and in combination and evaluated model performance using cross-validated prediction error [ΔMSE (mean squared error); see Materials and Methods]. Inclusion of lick rate substantially improved model performance for both SPN subtypes (dSPN: ΔMSE = −0.0427 [−1.250, −0.0034]; iSPN: ΔMSE = −0.0198 [−0.516, −0.0005]), whereas the addition of the withholding regressor alone produced only minimal improvements (dSPN: ΔMSE = −0.0004 [−0.0024, 0.0008]; iSPN: ΔMSE = −0.0003 [−0.0021, 0.0005]). The full model including both predictors yielded the best predictive performance (dSPN: ΔMSE = −0.0429 [−1.260, −0.0038]; iSPN: ΔMSE = −0.0200 [−0.521, −0.0005]) while eliminating the late divergence between Go and NoGo sound kernels (Fig. 3, K to L, and fig. S5, B and D). These results suggest that a late component of the NoGo sound-triggered Ca^2+^ signal in iSPNs reflects neural recruitment associated with inhibitory control. To illustrate this more directly, we plotted the partial model predictions for sound instruction, response withholding, and licking throughout the 1.15-s response window for both Go and NoGo trials (Fig. 3M). Consistent with our interpretation, we found that both reduced lick rate and active withholding were associated with persistent increases in iSPN signal throughout the NoGo response window (Fig. 3M, right), reflecting sustained iSPN activity throughout the response window on Correct Rejection trials. On Go trials, the onset of licking was accompanied by termination of the “withholding” signal and transition to negative lick signal in iSPNs, suggesting that the suppression of iSPN activity could permit “lick” expression (Fig. 3M, left). In contrast, partial predictions for dSPNs showed no clear relationship with lick rate across the response window (fig. S5C).

To see how TS SPN neural signals evolved throughout Go/NoGo training, we used a similar analytical framework for photometry recordings from the first day of NoGo exposure (fig. S6). As in the expert sessions, trial onset and sound presentation reliably evoked phasic recruitment of both SPN subtypes (fig. S6, A and B). In contrast to the expert sessions, recruitment on sound onset was qualitatively greater during NoGo trials (correct rejection and false alarm) than on Go trials (Hits) for both subtypes (fig. S6, A and B). Given the novelty of the NoGo sound, but not the go sound, on the first day of Go/NoGo training, this trend could reflect response to stimulus novelty more so than sound-related inhibitory control per se.

Notably, this trend only reached significance on comparison of raw hit and false alarm–evoked peaks in iSPNs (fig. S6, C and D) and in comparison of the early component of iSPN NoGo kernels with Go kernels (fig. S6, G and H). For the later window of the response period, the conclusions appeared similar to expert mice, with iSPNs again exhibiting a persistent Ca^2+^ signal through the response window that related to lower lick rate and active lick suppression (fig. S6, I, J, L, and M). We did not observe significant differences in sound-evoked activity based on sound frequency (fig. S6, K and L). Together, our population imaging highlights a complex SPN encoding of our task including: (i) an early tone-triggered activity component that does not distinguish Go and NoGo trials, seen in both SPN subtypes of expert mice; (ii) a later tone-triggered component of the response window in which iSPNs exhibit a persistent Ca^2+^ activity specifically during active witholding of NoGo trials.

### Isolated TS dSPN manipulations minimally impact sound-driven behavior

To examine how these SPN subtype specific patterns could contribute to auditory driven action control, we used cell type–specific optogenetic inhibition and excitation (fig. S6). On the basis of previously described roles of dSPNs in behavior and our photometry data showing enhanced recruitment of dSPNs on response (hit and false alarm) trials in expert mice, we wondered whether inhibition of dSPNs during target sounds would decrease hit trials and whether inhibition during nontarget sounds would decrease false alarms. We gained inhibitory optogenetic control of dSPNs by crossing D1Cre and Ai40D mice, which allow Cre-dependent expression of the inhibitory opsin ArchT3.0 (fig. S7A). The resulting fluorescence patterns was consistent with previously reported dopamine D1 receptor expression patterns and demonstrated robust substantia nigra fluorescence from axonal projections (fig. S7A). D1-Cre; R26R^AI40/+^ mice and their littermate controls (R26R^AI40/+^) were bilaterally implanted with fiber optic cannulae in TS and SPNs were inhibited during sound presentation on a randomized subset (25%) of Go/NoGo trials using 250 ms of 10 mW, 532-nm continuous green light stimulation (fig. S7, B and K). Unexpectedly, we found that dSPN inhibition specifically at target sound presentation did not alter the proportion of hit trials nor shift the distribution of hit reaction times (fig. S7, C, E, and L). Furthermore, the inhibition of dSPNs during nontarget sounds did not decrease the false alarm (FA) rate, although a floor effect could be possible (fig. S7C). We also observed a modest increase in FA reaction times and small decrease in response bias (fig. S7, D, E, and L).

We next tested whether dSPN excitation was sufficient to alter behavior (fig. S7, F to J and M). We expressed the red-shifted excitatory opsin, ChrimsonR in dSPNs via injection of a Cre-dependent AAV construct (AAV8-hSyn-FLEX-ChrimsonR-tdTom) with bilateral fiber optic cannula implants (fig. S7F). We selectively activated dSPNs during sound presentation of randomly selected Go/NoGo trials (25%) using 5 mW, 625-nm red, 20-Hz pulsed light (fig. S7G). The high hit rate in our task precluded strong conclusions about the ability of dSPN to increase responding to target tones, but excitation did produce a small but reliable reduction in hit reaction times (fig. S7, H, J, and M). Unexpectedly, the addition of dSPN activity during nontarget tones did not increase false alarms or reaction times (fig. S7, H, J, and M). Moreover, overall *d*′ and response bias did not significantly change during D1-SPN activation (fig. S7I). Together, these modest behavioral effects for dSPN perturbations may suggest the involvement of coordinated SPN subtype activity in reward-related responding, interference from concurrent lateral inhibition of iSPNs, or redundancy in the locus of behavioral control for reward-related responding (*26*, *59*). These findings underscore that greater phasic dSPN recruitment, as observed during Lick trials relative to No Lick trials, is not sufficient to bias behavior in this task.

### Perturbations of TS iSPN activity alters sound-driven action control

**Fig. 4.**
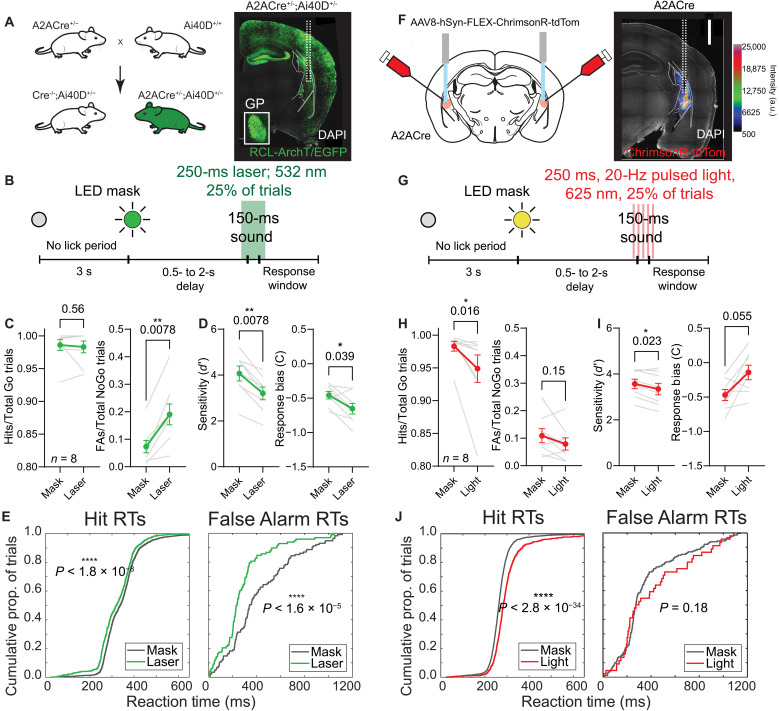
The indirect pathway mediates correct rejection of non-target stimulus. (**A**) Breeding strategy to express ArchT in iSPNs (scale bar, 1 mm); inset shows terminal expression in globus pallidus. Illustration adapted from SciDraw under CC-BY 4.0 license (https://creativecommons.org/licenses/by/4.0/; mouse, E. Tyler and L. Kravitz, DOI: 10.5281/zenodo.3925901). (**B**) Schematic of laser-mediated inhibition during sound presentation. Sound instruction mapping was counterbalanced. (**C** and **D**) Behavioral performance during mask control and laser inhibition trials (*n* = 8; means ± SEM). (C) Hit rate unchanged (*W* = 7, *P* = 0.56), false alarm rate increased (*W* = 36, *P* = 0.0078). (D) Discrimination (*d*′) decreased (*W* = −36, *P* = 0.0078) and response bias decreased (*W* = −30, *P* = 0.039). (**E**) Cumulative reaction time (RT) distributions for hits and false alarms pooled across sessions (mask: *n*_hit_ = 2447, *n*_FA_ = 115; laser: *n*_hit_ = 720, *n*_FA_ = 98). Kolmogorov-Smirnov tests: Hits, *D* = 0.13, *P* = 1.8 × 10^−8^; False Alarms, *D* = 0.33, *P* = 1.6 × 10^−5^. (**F**) Strategy to express ChrimsonR in iSPNs. Scale bar, 1 mm. Atlas diagrams used with permission of Elsevier Science & Technology Journals, from “*The Mouse Brain in Stereotaxic Coordinates*” by G. Paxinos and K. B.J. Franklin, second edition, 2001 (permission conveyed through Copyright Clearance Center Inc). (**G**) Schematic of light-mediated excitation during sound presentation. (**H** and **I**) Behavioral performance during mask and light excitation trials (*n* = 8; means ± SEM; gray lines connect mice). (H) Hit rate decreased (*W* = −34, *P* = 0.016), false alarm rate unchanged (*W* = −22, *P* = 0.15). (I) Discrimination (*d*′) decreased (*W* = −32, *P* = 0.023); response bias unchanged (*W* = 28, *P* = 0.055). (**J**) Cumulative RT (mask: *n*_hit_ = 2525, *n*_FA_ = 831; light: *n*_hit_ = 172, *n*_FA_ = 44). Kolmogorov-Smirnov tests: Hits, *D* = 0.25, *P* = 2.8 × 10^−34^; False Alarms, *D* = 0.18, *P* = 0.18.

In our photometry experiments, we observed robust phasic recruitment of iSPNs across trial types at sound onset, hinting at an important role for iSPNs in auditory-driven response control. To interrogate the behavioral effects of this sound-related activity across trial types, we inhibited iSPN activity during sound presentation. On the basis of previously described roles of iSPNs in behavior, we hypothesized that this would increase the proportion of false alarm responding (Fig. 4, A to E). We crossed A2A-Cre and R26R^Ai40D^ mice and observed robust ArchT3.0 expression of TS iSPNs in addition to some non-neuronal labeling in cortex and hippocampus (Fig. 4A and fig. S8A). We did not observe any differences in behavioral performance between our D1-ArchT and A2A-ArchT transgenic strains (fig. S3, E to H). When we randomly inhibited iSPNs during nontarget sound presentation, we found a robust increase in false alarm rate, which was matched with a decrease in reaction time to the NoGo stimulus (Fig. 4, C and E, and fig. S8B). While the inhibition of iSPNs during target sounds did not change the hit rate, we did observe a consistent reduction in the reaction time associated with target responding (Fig. 4E and fig. S8B). Overall, impaired NoGo performance was associated with a significant reduction in discriminative sensitivity, accompanied by an increased bias toward licking responses (Fig. 4D). This pattern suggests a shift toward a more impulsive response strategy, in which animals were less likely to withhold licking in the presence of the NoGo stimulus. No light related effects were observed in Cre-negative controls (fig. S9, A to D and H).

Our photometry data additionally suggested that sustained iSPN activity following sound presentation is associated with response withholding. To probe the temporal constraints of iSPN function, we performed another 250-ms iSPN inhibition after the sound presentation, timed to the average onset of the lick response (fig. S8C). We found that this temporally delayed manipulation similarly reduced false alarms and accompanying signal detection metrics while having no impact on response times for either trial type (fig. S8, D to G). Once again, there were no robust light-related effects observed in Cre-negative controls during post-sound sessions (fig. S9, E to G and I). These findings provide additional support for the idea that sustained iSPN drive following sound presentation could facilitate response withholding.

Last, we tested the impact of ectopically activating iSPNs bilaterally using of the excitatory red-shifted channelrhodopsin variant ChrimsonR (AAV8-hSyn-FLEX-ChrimsonR-tdTom injected in A2a-Cre mice; Fig. 4, F and G). We hypothesized that augmenting iSPN activity could decrease FA and target sound responding, promoting a more conservative response bias. We found that excitation of iSPNs during Go trials significantly decreased the hit responses and increased reaction times (Fig. 4, H and J). We did not observe a significant change in false alarm rate or false alarm reaction time with iSPN activation during the NoGo stimulus. This selective impairment in the hit rate resulted in a small but consistent decrease in sensitivity and a trending increase in response bias consistent with a reduced bias toward licking (Fig. 4I). Together, these data suggest that iSPN activity mediates response withholding for nontarget sounds and can support more conservative response strategies to sounds.

### Optogenetic manipulation of TS iSPNs biases licking after sound-action association forms

To examine whether our iSPN manipulations are modulating licking as a learned behavioral response or directly triggering motor output, we manipulated iSPNs outside the direct context of our behavioral paradigm (fig. S10). We examined two training timepoints—the first occurring before animals had ever undergone any auditory training (Naïve Opto), and the second session occurred after animals had learned to successfully restrict their lick responses to the Go sound (Go Trained Opto) (fig. S10, A and B). In these sessions, auditory stimuli were omitted entirely, and licking did not influence reward delivery. To encourage free licking, there were blocks of trials during which water was randomly dispensed (fig. S10B). These blocks were then followed by stimulation blocks, where 1 s of light stimulation randomly occurred. Licking during these blocks were recorded and analyzed to determine how SPN stimulation affects free licking probability.

When animals were naive to any type of sound training, iSPN stimulation did not alter free licking rate (fig. S10, C, D, G, and H). However, after animals had learned to restrict their licking to be used as a response to the Go stimulus, iSPN manipulation demonstrated opponent effects on free licking rate. iSPN excitation significantly decreased the lick rate, whereas iSPN inhibition significantly increased the lick rate (fig. S10, E and I). Notably, these changes in lick rate were not consistently observed across trials, as even animals displaying the strongest phenotype did not show reliable alterations in licking patterns indicative of direct motor program execution (fig. S10, F and J). Therefore, it seems that indirect pathway neurons can bidirectionally bias free licking probability even in the absence of sound. However, this only occurs after animals have been trained to use the act of licking as a sound-driven behavioral response.

### Neurexin1α-associated impairments in recruitment of TS iSPNs and inhibitory control

Thus far, our circuit analyses suggest that impairments of iSPN recruitment within the TS could disrupt the ability to inhibit responses to nontarget stimuli. We next sought to investigate whether synaptic dysfunction within this circuit could contribute to the inhibitory control impairments frequently seen in NDDs using a genetic model with previously established iSPN corticostriatal alterations. Nrxn1α is a synaptic adhesion molecule whose disruption is associated with a range of neurodevelopmental and psychiatric disorders including autism, Tourette syndrome, schizophrenia, and attention-deficit hyperactivity disorder (*46*, *50*–*57*, *60*, *61*). Prior acute slice studies have shown that dorsomedial prefrontal cortical neurons in both Nrxn1α^+/−^ and Nrxn1α^−/−^ mice exhibit a reduction in excitatory synaptic strength for connections onto iSPNs, but not dSPNs of the DMS (*46*).

**Fig. 5.**
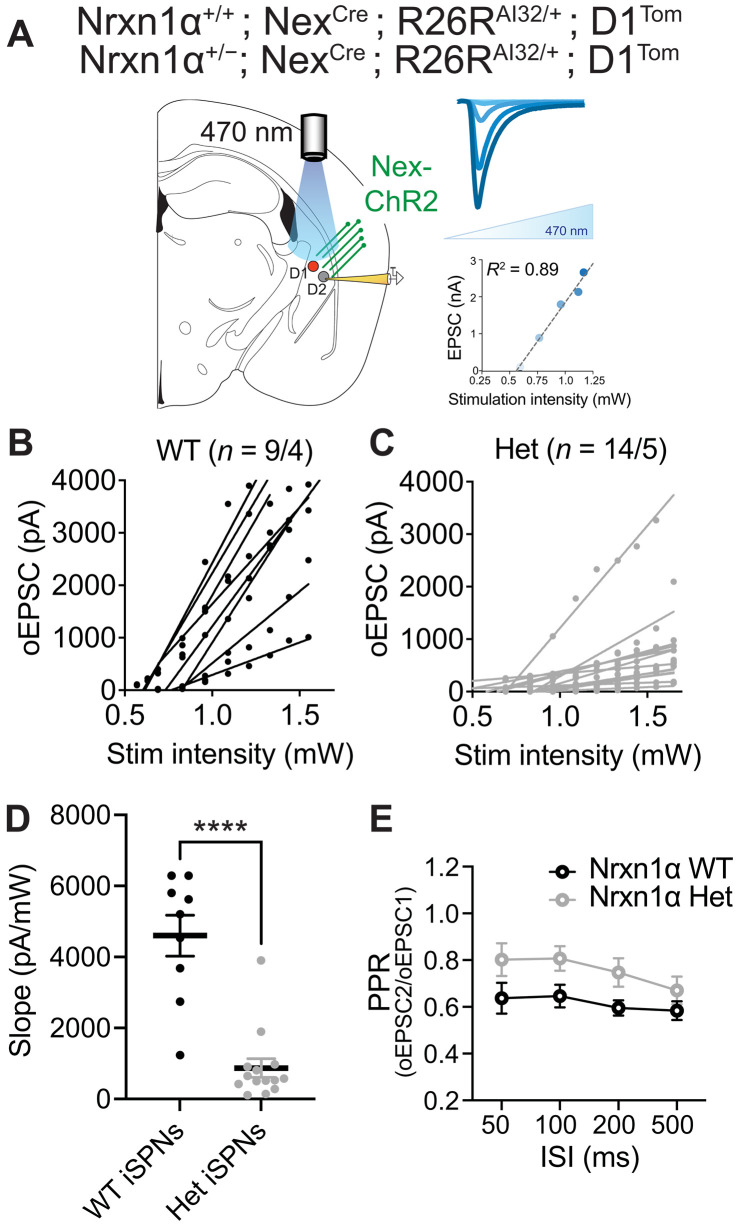
Nrxn1α mutant mice exhibit impaired cortical recruitment of iSPNs within the TS. (**A**) Experimental strategy to generate Nrxn1α transgenic mice expressing tdTomato in dSPNs as well as channelrhodopsin2 (ChR2) in cortical inputs. Patched iSPNs were identified as tdTomato negative SPNs. Right, top: Inward excitatory synaptic currents increased with greater light intensity, and synaptic strength could be approximated from a linear fit (right, bottom). Atlas diagrams used with permission of Elsevier Science & Technology Journals, from “*The Mouse Brain in Stereotaxic Coordinates*” by G. Paxinos and K. B.J. Franklin, second edition, 2001 (permission conveyed through Copyright Clearance Center Inc). (**B**) Optically evoked excitatory postsynaptic currents (oEPSC) linear fits for iSPNs from Nrxn1α WT mice (*n* = 9 cells from 4 mice). (**C**) oEPSCs linear fits for iSPNs from Nrxn1α heterozygous mice (*n* = 14 cells from 5 mice). (**D**) Comparison of the slopes from the fits shown in (B) and (C). Individual slopes represented by dots with group means ± SEM shown. Welch’s two-sample *t* test (two-sided): WT (*n* = 9, *x¯* = 4600) versus Het (*n* = 14, *x¯* = 873), *t*_11.3_ = 5.87, *P* < 0.0001****; Δ(Het − WT) = −3728 ± 636 SEM; 95% confidence interval [−5122, −2333]. (**E**) Paired-pulse ratios (PPRs) for optical stimulation of iSPNs from Nrxn1α WT versus Nrxn1α heterozygous mice across increasing interstimulus intervals (ISIs). Two-way analysis of variance (ANOVA) (ISI, genotype). Genotype effect: *F*_1,84_ = 11.82, *P* = 0.0009; no ISI effect: *F*_3,84_ = 1.29, *P* = 0.28; no interaction: *F*_3,84_ = 0.20, *P* = 0.896.

We first investigated whether this synaptic deficit could also be observed in the TS. To quantitatively measure cortically derived synaptic strength to the tail, we aimed to express the excitatory opsin ChR within all excitatory cortical inputs in Nrxn1α mutant mice. To accomplish this, we took advantage of the previously described haploinsufficiency of Nrxn1α (Nrxn1α^+/−^ and Nrxn1α^−/−^ exhibit similar magnitude reductions in synaptic strength in DMS) (*46*), crossing Nrxn1α^+/−^ mice to Nex^Cre/Cre^; R26R^AI32/AI32^; D1-Tomato mice to generate either Nrxn1α^+/+^ or Nrxn1α^+/−^ mice with a single copy of both Nex^Cre/+^ and R26R^AI32/+^, a Cre-sensitive ChR134-EYFP reporter line (Fig. 5A). This allows us to systematically compare the strength of corticostriatal synaptic inputs from neocortex between Nrxn1α genotypes by leveraging a genetics-based normalization of channel-rhodopsin expression (*62*). Using fluorescent-guided patching of the D1-Tom allele, we recorded from putative iSPNs identified by the absence of tdTOM.

We recorded pharmacologically isolated excitatory synaptic currents in whole-cell voltage clamp configuration, subjected each slice to increasing light intensity to sequentially recruit a larger proportion of cortical inputs (Fig. 5A). We quantified this input-output function by fitting evoked currents to a linear model and reporting slope as a measure of synaptic strength. Consistent with prior work in DMS, we found that iSPNs in the TS had a markedly reduced synaptic strength slope in Nrxn1α^+/−^ mice as compared to Nrxn1α^+/+^ (Fig. 5, B to D). Furthermore, we found that the reduction in TS iSPN synaptic drive was accompanied by a broad increase in paired-pulse ratio, suggestive of a decrease in presynaptic release probability (Fig. 5E). These findings extend prior observations in the DMS to the striatal tail, demonstrating that Nrxn1α haploinsufficiency results in a robust reduction in cortical excitatory drive onto iSPNs throughout the dorsal striatum.

**Fig. 6.**
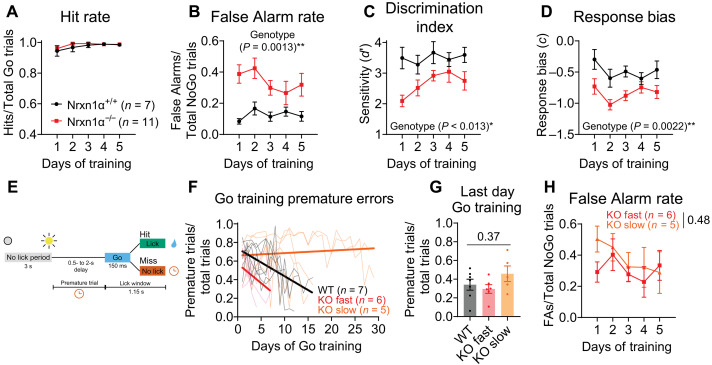
Nrxn1α homozygous KO mice exhibit deficits in withholding responses to nontarget sounds. (**A** to **D**) Behavioral performance of Nrxn1α WT (*n* = 7) and KO (*n* = 11) mice across Go/NoGo training (means ± SEM). Sound instruction mapping was counterbalanced. Data were analyzed using two-way mixed-effects models (REML) with fixed effects of training (day) and genotype and a random intercept for subject (Greenhouse-Geisser corrected). (A) Hit rate: effect of Training (*F*_1.996,30.44_ = 3.49, *P* = 0.043); no genotype effect (*F*_1,16_ = 0.47, *P* = 0.50) or interaction (*F*_4,61_ = 0.36, *P* = 0.84). (B) False alarm rate: Genotype effect (*F*_1,16_ = 15.25, *P* = 0.0013); no training effect (*F*_3.02,45.98_ = 1.21, *P* = 0.32) or interaction (*F*_4,61_ = 1.07, *P* = 0.38). (C) Discrimination (*d*′): Genotype effect (*F*_1,16_ = 7.898, *P* = 0.013); no training effect (*F*_3.26,49.77_ = 2.37, *P* = 0.077) or interaction (*F*_4,61_ = 1.595, *P* = 0.19). (D) Response bias: Genotype effect (*F*_1,16_ = 13.22, *P* = 0.0022); no training effect (*F*_3.17,48.26_ = 2.369, *P* = 0.079) or interaction (*F*_4,61_ = 0.69, *P* = 0.60). (**E**) Trial schematic for Go training. (**F**) Go training curves for WT, KO fast learners, and KO slow learners. (**G**) Premature error rates on the final Go training day (WT: *n* = 7; KO fast: *n* = 6; KO slow: *n* = 5; means ± SEM). Kruskal-Wallis test: *H* = 2.09, *P* = 0.37. (**H**) False alarm rates from (B) with KO mice split by Go learning group. Two-way mixed-effects model (REML) with training and learning group as fixed effects: no effect of training (*F*_2.74,22.60_ = 1.62, *P* = 0.21), learning group (*F*_1,9_ = 0.55, *P* = 0.48), or interaction (*F*_4,33_ = 0.82, *P* = 0.52).

Given the evidence that reductions in iSPN recruitment could increase inappropriate responding to nontarget sounds (Figs. 3 and 4), together with observations that cortical synaptic transmission to TS iSPNs is impaired (Fig. 5), we hypothesized that mice with mutations in Nrxn1α would show deficits in sound-driven response inhibition. To test this, we trained Nrxn1α^+/+^ [wild-type (WT)] mice and littermate Nrxn1α^−/−^ (KOs) mice in our Go/NoGo task to investigate any deficits in inhibitory control to the NoGo stimulus. Here, we elected to use the sensitized genetic background of Nrxn1α KOs rather than the more clinically relevant Nrxn1α^+/−^ model as prior mouse work has shown variable penetrance of numerous behavioral phenotypes for Nrxn1α (*63*). During Go/NoGo training, although KO mice demonstrated similar hit rates to their littermate controls, KOs exhibited greater false alarm rates throughout initial training (Fig. 6, A and B). This corresponded with a decrease in the discriminative performance for KOs and greater bias toward responding compared to WTs, regardless of the stimuli (Fig. 6, C and D). Response times were shorter in KOs than in WTs on Hit trials but did not differ significantly on False Alarm trials (cumulative distribution plots shown in fig. S11).

Although previous reports of hyperactivity in Nrxn1α KOs have been inconsistent (*58*, *64*–*68*), it remains possible that a general increase in impulsive licking, rather than a sound-specific deficit in response inhibition, is driving the elevated false alarm rate observed in these mice. To examine this further, we investigated premature responses arising during the earlier Go training stage (Fig. 6E). Sound delivery in our task was predicated on a variable 0.5- to 2-s window of no licking following trial start, so we decided to use the number of violations of this rule as a proxy for global impulsivity. To reach criteria during the Go training stage, animals were required to reduce premature responses to achieve a *d*′ > 2 (comparison of hit to premature response rate). We therefore first examined whether Nrxn1α KO mice required more training sessions to reach criterion performance compared to WT controls. While some Nrxn1α KO mice required more sessions to reach criterion than their WT littermates, approximately half reached expert performance more quickly (Fig. 6F). To capture this variability, we classified KO animals as “fast” or “slow” learners based on whether they reached criterion in fewer than 12 sessions (Fig. 6F). Regardless of learning rate, as a group, Nrxn1α KO mice did not demonstrate significant differences in premature error rates upon reaching expert criteria (Fig. 6G). Next, we examined whether these fast and slow Go learners within the Nrxn1α KO group differed in their NoGo response inhibition, as fast Go learners might exhibit performance more comparable to WT controls. When we analyzed the proportion of false alarms for these two groups during the final stage of Go/NoGo training, we found that the KO groups were not statistically different in their learning, showing a similar time course and magnitude of increased false alarm rates (Fig. 6H). Together, these data suggest that while a subset of Nrxn1α KO mice exhibit generalized motor impulsivity, the entire KO cohort demonstrate a specific impairment in withholding responses to nontarget sounds.

### Increasing TS iSPN excitability can suppress Neurexin1α-associated sensory-motor impulsivity

**Fig. 7.**
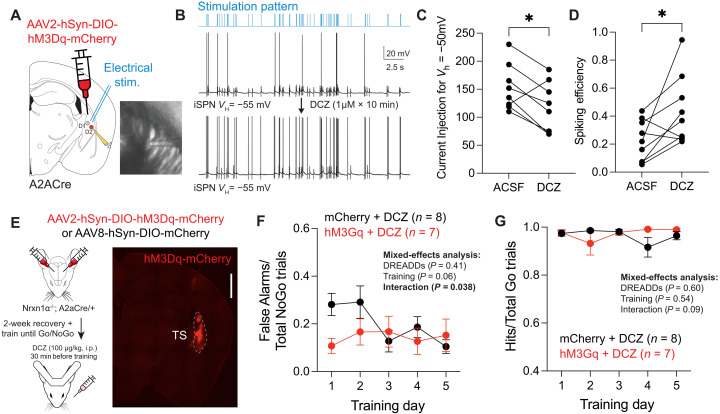
Chemogenetic recruitment of indirect pathway SPNs rescues sensorimotor impulsivity in Nrxn1α KO mice. (**A**) Experimental strategy used to test the ability of DCZ to increase excitability of iSPNs expressing the excitatory DREADD hM3Dq. iSPNs (*N* = 9 cells from 6 A2ACre animals injected with AAV2-hSyn-DIO-hM3Dq-mCherry) were recorded in whole-cell current clamp configuration, while local electrical stimulation was delivered to recruit excitatory cortical and thalamic afferents. Illustration adapted from SciDraw under CC-BY 4.0 license (https://creativecommons.org/licenses/by/4.0/; mouse head schema, L. Petrucco, DOI: 10.5281/zenodo.3925903). Atlas diagrams used with permission of Elsevier Science & Technology Journals, from “*The Mouse Brain in Stereotaxic Coordinates*” by G. Paxinos and K. B.J. Franklin, second edition, 2001 (permission conveyed through Copyright Clearance Center Inc). (**B**) Example responses of an iSPN held at −55 mV (*V*_H_ = holding potential) to a physiologic stimulation pattern before (top) and 10 min after (bottom) bath application of DCZ (1 μM). (**C**) DCZ significantly reduced the holding current (pA) required to maintain iSPNs at −50 mV and (**D**) increased spiking efficiency during local field stimulation (spikes per stimulation pulse). *P < 0.05. (C) *P* = 0.0223 from paired *t* test (*t* = 2.921, df = 7, *N* = 8). (D) *P* = 0.0335 from paired *t* test (*t* = 2.562, df = 8, *N* = 9). (**E**) To chemogenetically recruit iSPNs in Nrxn1α KO mice, Nrxn1α^−/−^; A2ACre/+ mice were bilaterally injected with AAV2-hSyn-DIO-hM3Dq-mCherry (*N* = 7 animals) or mCherry control virus (*N* = 8 animals). A representative injection site is shown (right). Scale bar, 1 mm. Animals received an intraperitoneal injection of DCZ (100 μg/kg) 30 min before training beginning on the first day of Go/NoGo training. Mapping of high- and low-frequency band-limited sounds to Go and NoGo instructions was counterbalanced across animals. (**F**) Chemogenetic recruitment of iSPNs in Nrxn1α KO animals reduced false alarm rates early during training relative to KO animals expressing the control construct. (**G**) Hit rates were unaffected.

Given the broad expression of Nrxn1α protein throughout the brain, it is challenging to assess whether the impaired recruitment of TS iSPNs directly contributes to the increased false alarm responding observed in KO mice. To more directly interrogate this possibility, while also testing a potential circuit-based ameliorative strategy, we tested whether boosting the excitability of TS iSPNs was sufficient to suppress the increased false alarm responding of Nrxn1α KOs. To first probe the efficacy of this approach, we injected an excitatory DREADDs (designer receptors exclusively activated by designer drugs) construct, AAV2-hSyn-DIO-hM3D-mCherry, into the TS of A2a-Cre mice. After 3 weeks, we cut acute slices of TS and patched mCherry-positive iSPNs in whole-cell current clamp configuration (Fig. 7A). We used a previously validated assay to examine recruitment efficiency of TS by excitatory inputs—in short, we held TS iSPNs at −50 mV via current injection and quantified the proportion of action potentials evoked by complex stimulation patterns of local excitatory axons (Fig. 7B), first in artificial cerebrospinal fluid (ACSF) and then following 10 min of 1 μM deschloroclozapine (DCZ; a potent and specific hM3D ligand) exposure. In addition to noting a requirement for less current injection following DCZ (Fig. 7C; consistent with a DCZ-dependent depolarization of iSPN resting membrane potentials), we also observed a reliable increase in the spiking recruitment following DCZ incubation (Fig. 7D).

To test whether our chemogenetic strategy was effective in ameliorating the sensory-motor impulsivity of Nrxn1α KOs, we split a cohort Nrxn1α^−/−^; A2a-Cre mice, bilaterally injecting the TS with either AAV2-hSyn-DIO-hM3D-mCherry or an empty mCherry control (Fig. 7E). Following 2 weeks of recovery, we trained mice on Go training. We did not observe a significant difference in training timeline between groups [mCherry: mean(days) = 13.4, SD(days) = 7.6, *N*(animals) = 8; hM3Dq: mean(days) = 15.9, SD(days) = 6.5, *N*(animals) = 7; Student’s *t*(13) = 0.67, *P*(two-tailed) = 0.51]. We then delivered DCZ (100 μg/kg) via intraperitoneal injection 30 min before each day of full Go/NoGo training (Fig. 7E). We found that this intervention suppressed the proportion of false alarm trials, particularly in the first 2 days of NoGo sound exposure (Fig. 7F), leading to improvements in *d*′ over Nrxn1α KOs that only expressed the control virus. Furthermore, we observed that enhancing iSPN excitability did not have impacts on correct responding to the target tone (Fig. 7G). Together, these data suggest that boosting excitability of TS iSPNs is sufficient to partially reduce the false alarm responding seen in Nrxn1α KO mice, offering a putative circuit target for sensory-motor impulsivity associated with NDDs.

## DISCUSSION

In this study, we used an auditory Go/NoGo task to examine the striatal circuitry supporting inhibitory control for sensory-driven behavior, with a particular focus on how disruptions of these mechanisms may contribute to inhibitory control impairments in a genetic NDD model. We found that while both SPN subtypes were robustly recruited by both Go and NoGo sounds early in the response window, only iSPNs had persistent activity on NoGo trials, consistent with a unique mechanistic role in suppressing responses to nontarget stimuli. Optogenetic manipulations of iSPNs during sound presentation bidirectionally altered lick responses consistent with their canonical roles in action suppression (*69*). In addition, iSPN inhibition following sound presentation impaired response withholding on NoGo trials, hinting at a potential role of sustained iSPN activity in sound-driven response withholding. Nrxn1α-mutant mice, a genetic model for multiple NDDs, exhibited corticostriatal synaptic deficits onto iSPNs in the TS while demonstrating an impaired ability to withhold responses to nontarget stimuli, consistent with the role we uncovered for TS iSPNs in mediating response control. Chemogenetically enhancing the excitability of TS iSPNs in these Nrxn1α mutant mice ameliorated these deficits in inhibitory control, offering a putative circuit target for sensorimotor impulsivity in disease.

### iSPNs in the TS contribute to inhibitory control over sensory-driven action

One of central findings of this work is that TS SPN subtypes differentially contribute to behavioral control during the presentation of nontarget (i.e., NoGo) auditory stimuli. The most obvious differences seen between the Ca^2+^ activity of SPN subtypes imaged at the population level was found in the latter half of the response window (∼0.58 to 1.15 s), where iSPNs exhibited a persistent signal on No-Go trials that was not found in dSPNs (Fig. 3I). Inclusion of two motor-related parameters in our encoding model, one capturing lick-rate and another capturing the response-withholding process, accounted for the trial type differences seen in iSPNs (Fig. 3, K to M), suggesting that persistent iSPN activity following sound presentation could support the sensory-evoked motor suppression. Given the limitations of photometry in resolving distinct neuronal subpopulations and the strong sound-evoked responses in the early response window (0 to 0.58 s), it is difficult to say whether there is earlier sound-triggered iSPN activity that could also support response suppression (however, see below). In addition, it is possible that the signals we record here reflect dendritic excitatory drive onto SPNs rather than SPN firing per se, given recent findings suggesting that SPN photometry signals reflect nonsomatic Ca^2+^ levels (*70*). However, other work has shown that axonal SPN photometry signals in downstream substantia nigra reticulata strongly correlate with striatal SPN photometry signals (*71*), suggesting robust representation of somatic activity. Regardless of the specific interpretation, our results suggest that increased iSPN recruitment is associated with sensory-driven response withholding.

Consistent with the iSPN photometry, our optogenetic inhibition of iSPNs during sound presentation supports a role for iSPNs in inhibitory control of sound-driven responses to inappropriate or unfavorable sensory stimuli. We show that iSPN inhibition starting either 50 ms before sound presentation or from 200 to 450 ms after sound presentation both increase the proportion of false alarms, with only the early manipulation biasing toward faster response times in both Hit and False Alarm trials. These data provide strong functional support for this persistent activity but suggest an earlier temporal window for iSPN control of lick timing. Despite the brief 150-ms temporal windows used for inhibition, it remains unknown how long the network effects of our manipulations last. Hence, it is difficult to know whether the early iSPN manipulation phenotype arises from disruption of another inhibitory control subpopulation intermingled with iSPNs encoding basic auditory features or whether the manipulation simply impairs the persistent activity that we resolve in the late response window. The optogenetic excitation of iSPNs significantly reduced hit rates, in keeping with the hypothesis that iSPNs constrain operant responding. However, we unexpectedly did not observe a reduction in false alarm rates. We believe this likely constitutes a floor effect: some base level of false alarm rates appears to feature of the strategy with which most of our mice solve this task to maximize reward and might also be shaped by the relative preponderance of Go trials (60%) relative to NoGo trials (40%) in our task design.

These findings are in line with previous reports in other striatal regions associating iSPN activity with the inhibition of unrewarded actions and subsequent facilitation of exploratory behavior (*69*, *72*). Moreover, these cell type–specific manipulations extend previous work in nonhuman primates, implicating that globus pallidus externa projecting neurons from the caudate tail are involved in the rejection of low-value visual stimuli (*40*, *41*). Together, these data support proposed evolutionary homologies between the rodent TS and the primate caudate tail (*32*–*34*) and provide mechanistic insight into indirect pathway involvement in inhibitory control*.* Furthermore, they provide further circuit-level mechanistic insights into the attention-like properties of sensory selection (*73*, *74*) that the striatum contributes to, highlighting the key role that iSPNs can play in restricting motor response to nontarget tones. Further behavioral work is needed to determine whether these same circuits are used for filtering irrelevant, distracting stimuli. In addition, further work could investigate the extent to which these roles of iSPNs are restricted to the striatal tail. Sensorimotor integration is likely distributed across the anteroposterior striatal axis, possibly depending on the sensory modality in question (*69*). For example, in a tactile Go/NoGo task, one might expect comparable effects with iSPNs in more anterior and lateral striatal territories, in which the whiskers and face are more abundantly represented (*13*, *14*).

We also demonstrated that manipulation of iSPNs in naïve mice does not bias free-licking behavior, supporting previous work proposing that this striatal region does not directly mediate general motor execution but rather contributes to perceptual decision-making (*26*, *37*, *75*). Furthermore, recent work has proposed a particular involvement of the TS in facilitating sound-action associations, with dopamine signaling correlating to the reliability of a given action in a specific sensory context (*38*). This is further consistent with literature implicating the TS in fear conditioning, during which a previously neutral stimulus comes to elicit a conditioned freezing response (*32*, *39*, *76*). Our findings that iSPNs are sufficient to bias free-licking behavior in the absence of auditory stimuli, but only after animals have been trained to organize licking as a response to the Go stimulus, support a role for the TS in regulating learned stimulus-action associations.

Particularly important points for future consideration are the potential mediators of the persistent iSPN activity within TS that contributes to sensory-mediated inhibitory control. Given that the striatum typically reflects an integration of its broad cortical and thalamic excitatory drivers, top candidates include auditory and prefrontal cortices or the medial geniculate body. Prior work has highlighted important behavioral effects for each of these inputs to TS (*37*, *77*), but further work is required to understand the specific contributions to auditory-driven sensory inhibition. Given the SPN subtype specificity of the persistent activity, another potential mediator could be reduced feed-forward inhibition, as recent work has highlighted the strong inhibitory impact of fast-spiking interneurons selectively onto TS iSPNs (*78*).

### Coordinated versus independent effects: The contributions of the TS direct pathway

A puzzling result of our study was the absence of robust functional effects for TS dSPNs, either via optogenetic excitation or inhibition. This result was especially surprising in light of our photometry data, which showed greater sound-evoked recruitment of dSPNs on Lick compared with No Lick trials. Given the verification of our tools and the robust behavioral effects observed with iSPN manipulations, we do not believe these results represent technical issues. Instead, we must consider two alternatives: (i) dSPNs work in conjunction with iSPNs for target responding or (ii) target-responding behavioral control has moved to another circuit outside of TS. The analysis of the raw photometry data is suggestive of a causal role for dSPNs in lick responding, as hit and false alarms appear to have larger signal than correct rejections (Fig. 3, C and E), a pattern that was only weakly captured in iSPNs (Fig. 3, D and F). Work in other striatal areas has demonstrated that concurrent activation of both striatal pathways is necessary for the initiation of actions (*79*–*81*), with widely accepted models of striatal function proposing that concurrently active iSPNs contribute to action control by suppressing alternative actions or by mediating the required timing and synchrony of striatal activity to initiate action (*79*, *82*–*86*). While we did not explicitly test this coordinated model for target-responding here, we can note that the simultaneous inhibition of both pathways that likely accompanies muscimol was effective in blunting reward-related responses. Nevertheless, a more targeted strategy should be used in the future to more precisely address this issue.

In addition, because the Go cue was trained before the NoGo cue was introduced, animals may treat NoGo trials as extinction of previously reinforced tone-evoked responding. Given canonical roles of iSPNs in action suppression (*69*, *87*, *88*), training order is therefore an important consideration when interpreting our results. Another important implication of the training order is that False Alarm responses early in NoGo training might constitute genuine mistakes as animals learn to disambiguate Go and NoGo sounds, rather than bona fide failures in response inhibition. To minimize this concern, photometry and optogenetic data shown here were collected from “expert” animals that had reached *d*′ > 2 on at least three consecutive Go/NoGo sessions. Nonetheless, this consideration remains relevant for the interpretation of our Nrxn1α data where key effects occur early in Go/NoGo training.

### Impaired recruitment of the TS indirect pathway: A potential circuit mechanism for sensory-motor impulsivity

Another central finding of this study is the identification of an Nrxn1α-related synaptic deficit in the corticostriatal inputs onto iSPNs in the TS. This was consistent with recent work demonstrating impaired synaptic strength in corticostriatal projections of the PFC to the anterior DMS in both Nrxn1α^+/−^ and Nrxn1α^−/−^ mice (*46*). These data imply that impaired excitatory synaptic iSPN recruitment is a hallmark of the striatum of mice with Nrxn1α disruptions, a circuit vulnerability that could lead to broadly dysregulated cortical activity through the basal ganglia loop architecture. A limitation of the Nex-Cre mediated optogenetic strategy is our inability to determine the specific cortical inputs most impacted by the loss of Nrxn1α function. The extent and specificity of Nrxn1α associated synaptic deficits may differ across distinct cortical projection populations, an important consideration when parsing the contributions of PFC and auditory cortical dysregulation to altered behavior. In contrast to corticostriatal inputs, prior work from our laboratory has shown that thalamic parafascicular inputs to the dorsal striatum do not exhibit deficits in striatal SPN recruitment in Nrxn1α KOs (*46*). While it is tempting to suggest this specifically implicates corticostriatal synaptic dysfunction in instances of altered behavioral control, further work is required to fully rule out thalamic contributions.

In keeping with an impaired recruitment of iSPNs within TS, we found that Nrxn1α KO mice showed a selective deficit in inhibitory control over nontarget stimuli, distinct from a general motor hyperactivity phenotype. Given the functional importance of TS iSPN activity identified in our study, it is possible that this synaptic deficit contributes to the observed learning impairments. To specifically test this, we used selective expression of an excitatory chemogenetic modulator in TS iSPNs of the Nrxn1α KOs (Fig. 7). We found that boosting the excitability of TS iSPNs increased their recruitment to excitatory synaptic input and was able to suppress the increased false alarm responding as compared to Nrxn1α KOs that only received control virus. These data permit several key conclusions: (i) it reinforces the general importance of TS in mediating sensory-driven motor inhibition; (ii) it demonstrates that the TS is a specific node of circuit dysfunction in the enhanced non-target responding of Nrxn1α KO mice; and (iii) it suggests that pharmacological approaches that can enhance TS iSPN recruitment are mechanistically valid potential therapeutic targets for motor impulsivity. Future work should examine whether impaired TS iSPN recruitment is a common circuit alteration in other ASD and ADHD genetic models.

Further support for our circuit-behavioral model of sensory-driven motor impulsivity come from similar NoGo learning impairments in Nrxn1α^−/−^ mice training in a visual Go/NoGo paradigm, recently reported in a publicly available doctoral dissertation, lending convergent validity to our findings across independent laboratories and sensory modalities (*89*). Our observed behavioral phenotype is in line with the clinical characterization of Nrxn1α-associated disorders, as impairments in sensory processing, sensorimotor gating, and inhibitory control are clinically relevant features in ASD, ADHD, Tourette’s syndrome, and schizophrenia (*89*–*95*). Furthermore, many of these disorders are highly comorbid with each other, suggesting commonalities in the underlying neural aberrations that give rise to these shared symptoms (*92*, *96*–*98*). Given the established association of Nrxn1α copy number variants with these NDDs (*51*, *99*), the circuit mechanisms identified in this study are likely to have clinical relevance and may represent a promising target for future therapeutic development.

## MATERIALS AND METHODS

### Animals

All procedures and experiments were conducted in accordance with the National Institutes of Health Guidelines for the Use of Animals and approved by the University of Pennsylvania Institutional Animal Care and Use Committee (protocol: 805643; confirmation number: aagehdb). All animals used in this study were adult mice (2.5 to 5 months). We used WT mice (C57BL/6J; the Jackson Laboratory, strain: 000664), heterozygous mice bred from the Adora2a-Cre line [B6.FVB(Cg)-Tg(Adora2a-cre)KG139Gsat/Mmucd], and heterozygous mice bred from the Drd1a-Cre line [B6;129-Tg(Drd1-cre)120Mxu/Mmjax; MMRRC_037156-JAX]. Homozygous Ai40D male breeders (B6.Cg-Gt(ROSA)26Sortm40.1(CAG-aop3/EGFP)Hze/J; IMSR_JAX:021188) were obtained from the Jackson Laboratories to cross with either heterozygous Adora2a-Cre or Drd1a-Cre mice to generate double-transgenic mice used for experiments. From these breedings, we used Adora2a-Cre heterozygous; Ai40d heterozygous double-transgenic mice and Drd1a-Cre heterozygous; Ai40d heterozygous double-transgenic mice. Constitutive Nrxn1α KO mice were originally obtained from the Südhof Lab and were maintained as previously described (*46*, *100*). For slice experiments, Nrxn1α^+/−^ breeders were crossed to Nex^Cre/Cre^;R26R^AI32/AI32^;D1-Tomato mice [Nex-Cre mice: obtained with permission from K.-A. Nave from the Zhou Lab, University of Pennsylvania; AI32:B6;129S-Gt(ROSA)26Sor-CAG-ChR2(H134R)-EYFP, JAX012569; D1-Tom: B6.Cg-Tg (Drd1a-tdTomato, JAX016204] to generate Nrxn1α^+/+^;Nex^Cre/+^;R26R^AI32/+^;D1^Tom^ or Nrxn1α^+/−^;Nex^Cre/+^;R26R^AI32/+^;D1^Tom^ experimental mice. Control animals for all experiments consisted of age- and sex-matched mixed littermates. All animals were housed with a 12-hour light/12-hour dark cycle with food provided ad libitum and a restricted water schedule. Animals were typically group housed (two to five per cage), with the exception of muscimol-treated cohorts and individuals displaying aggressive behavior toward cage mates.

### Behavioral apparatus and training

To assess value-based responses to auditory stimuli, we used a head-fixed auditory Go/NoGo behavior where licking was used to register a response. Behavioral apparatuses were custom-built within double-walled sound-attenuating chambers containing head-fixation clamps (ThorLabs), a three dimensionally printed platform (derived from a treadmill design by Janelia J005558), an optical lickometer (Sanworks 1020), trial start light-emitting diode (LED) (either white or green as it served as a mask for optogenetic experiments), water dispensing system, and sound system. Behavioral programs were operated using custom-built circuits with Arduino Uno and Teensy 3.2 microcontrollers and custom software. Behavioral data were acquired via serial communication and saved as CSV files using the CoolTerm terminal application.

Our water dispensing system consisted of a solenoid valve (The Lee Company LHDA1231115H) with tubing (Tygon S3 E-3603) and an elevated water reservoir (30-ml syringe; Thermo Fisher Scientific, 22124969). As water reward amount depended on the duration the solenoid valve was open and the height of the reservoir, the system was regularly calibrated to reliably dispense 4-μl reward volumes. Furthermore, water was replenished to the same height at the start of each session to maintain consistent fluid pressure.

Our sound system included an Adafruit Audio FX Sound Board (16 MB) connected to output speakers (DigiKey 668-1447-ND) for playback of bandlimited sound stimuli. These stimuli (5 to 7.2 kHz and 13.3 to 19.2 kHz) were generated using custom MATLAB code by applying calibrated bandpass filters to white noise, ensuring flattened spectral profiles across the target frequency ranges. Stimuli were calibrated to 65 decibels measured at the animal’s head position. To prevent spectral artifacts, all stimuli were onset- and offset-ramped using 5-ms raised cosine (cosine-squared) envelopes.

Before training, mice were water restricted to >85% of their initial body weight over the course of 2 to 3 days during which animals became familiar with experimental handling. Next, animals underwent one to two sessions of magazine training where mice acclimated to head fixation in the box by freely receiving reward. Animals then underwent two stages of behavioral shaping before reaching final Go/NoGo training. Mice were first trained to proficiency on the Go cue before being advanced to a phase that included both Go and NoGo cues. The assignment of auditory stimuli to Go and NoGo instruction was counterbalanced across cohorts. In half of the cohorts, the high band-limited sound (13.3 to 19.2 kHz) served as the Go cue, and the low band-limited sound (5 to 7.2 kHz) served as the NoGo cue; in the other half, the mapping was reversed. For all stages, each training session was ∼30-min long, and every trial began with a no-lick, dark period of three consecutive seconds before the box light turned on.

During initial association training, the Go sound was played for 1 s immediately before a randomly delivered reward (occurring 1 to 10 s after trial start). Animals were considered experts in this stage after training for 10 days or after receiving >2 rewards/min and demonstrating a significant increase in anticipatory licking during the cue compared to 1 s prior (for two consecutive days).

During the Go training stage, animals learned to lick within 1 s of the end of a 150-ms Go sound (total response window being 1.15 s). During this stage they also learned to withhold premature licking before the sound presentation. From this phase onward, a premature lick resulted in a 5-s lockout penalty. For initial Go training, the delay from trial start to Go sound presentation ranged from 0.5 to 1 s, and misses (defined as a failure to lick within 1 s after offset of the Go sound) were not punished. Once animals achieved a *d*′ ≥ 1.5 (calculated by comparing the hit rate and premature rate) for a session, animals progressed to the final Go training phase where the sound delay increased to up to 2 s, and misses were punished with a 5-s lockout penalty. Animals were considered experts at this stage once they reached a *d*′ ≥ 2 for three consecutive sessions.

Last, during Go/NoGo training, NoGo trials appeared randomly at a 40% probability during which animals received a 5-s lockout penalty for false alarm responses but avoided this penalty when correctly withholding their licking to the NoGo stimulus. As in Go trials, the total response window was 1.15 s for NoGo trials (false alarm, correct rejection). Animals were considered experts after reaching a *d*′ ≥ 2 for three consecutive sessions. During training sessions, incorrect trials were repeated; however, during all behavioral manipulation sessions, trials were presented in a fully random order.

### Behavioral analysis

For the analysis of behavioral data, we used custom MATLAB scripts. To quantify Go/NoGo performance, we used standard signal detection theory metrics. Discrimination index or *d*′ was quantified as *d*′ = *Z*(Hit Rate) − *Z*(False Alarm Rate) for the Go/NoGo stage and *d*′ = *Z*(Hit Rate) − *Z*(Premature Rate) for Go training stage. Response bias or the criterion metric was quantified as *c* = −0.5[*Z*(Hit Rate) + *Z*(False Alarm Rate)].

Intuitively, *d*′ reflects the animal’s ability to discriminate Go from NoGo stimuli, with higher values indicating better discrimination (i.e., high hit rates combined with low false alarm rates). In contrast, the criterion (*c*) reflects response bias, with negative values indicating a more liberal strategy (greater tendency to respond/lick) and positive values indicating a more conservative strategy (greater tendency to withhold responding).

Because *z*-transforms were undefined when hit or false alarm rates equal exactly 0 or 1, extreme rates were slightly adjusted according to the number of trials (Macmillan and Creelman correction), replacing 1 with (*n* − 0.5)/*n* and 0 with 0.5/*n*, where *n* is the number of relevant trials in that session.

As our paradigm does not require the animal to initiate trials, engagement was quantified during analysis by tracking the local miss rate and lick rate following reward. If the local miss rate rose past the engagement cutoff (or if animals stopped ingesting reward), then the remainder of the trials for that session were not analyzed. Engagement cutoffs were only used to calculate performance during training as well-trained animals typically maintained their engagement throughout the entire 30-min behavioral sessions.

### Surgical procedures

All surgical procedures were performed on a stereotaxic frame (Kopf Instruments, Model 1900). Briefly, mice were anesthetized using vaporized isoflurane (1 to 2% + oxygen at 1.5 liter/min). Body temperature was continuously monitored and maintained at 30°C during surgery (Harvard apparatus, #50722F; 55-7030). After the administration of ophthalmic ointment (Puralube Vet Ointment) and analgesics (Meloxicam-SR; bupivacaine), the scalp was prepared for surgery by removing overlying fur with depilatory cream, followed by alternating washes with 70% ethanol and betadine. A small anterior-posterior incision was made using a scalpel to expose the skull, which was then crosshatched with scalpel marks for texturization and cleaned with hydrogen peroxide. Bilateral craniotomies for injections and fiber implantations were made by drilling small (0.5 mm) holes above the target coordinates for the TS [anterior-posterior: +2.15 to 2.2 mm from the interaural line (−1.7 mm from bregma), medial-lateral: +/−3.5 mm, dorsal-ventral: −3.0 mm from cortical surface for injections, −2.6 to −2.8 mm for fiber placements].

Injections were performed using the Nanoject II/III system and a pulled glass needle backfilled with mineral oil. A total volume of 350 to 368 nl of virus was injected at a rate of ∼1 nl/s per injection site. Following each injection, the glass needle remained in place for about 10 to 15 min before being slowly withdrawn out to minimize backflow. For optogenetic excitation experiments, we used AAV8-hSyn-FLEX-ChrimsonR-tdTomato (UNC Vector Core) at a titre of 3.7 × 10^12^ vector genomes (V.G.)/ml. For photometry experiments, we used pGP-AAV9-syn-FLEX-jGCaMP8m-WPRE (Addgene) with an original titre of 2.4 × 10^13^ V.G./ml diluted to 4.8 × 10^12^ V.G./ml. For chemogenetics experiments, we used AAV2-hSyn-DIO-hM3Dq-mCherry (UNC Neurotools) with a titer of 8.39 × 10^12^ or control virus (AAV8-hSyn-DIO-mCherry; Addgene, original titer of 2 × 10^13^, diluted to 8 × 10^12^) injected bilaterally to 250 nl per hemisphere.

For all optogenetic experiments, we used fiber optic cannulae (RWD; R-FOC-BL200C-50NA) with white/black Φ1.25-mm ceramic ferrules with 0.5 numerical aperture (ΝΑ), 200-μm fiber optic cores at a length of 4 mm. For all photometry experiments, we used fiber optic cannulae (RWD; R-FOC-BL200C-39NA) with black Φ1.25-mm ceramic ferrules with 0.39 ΝΑ, 200-μm fiber optic cores at a length of 4 mm. Fibers were slowly lowered into the open craniotomies and secured in place with dental cement (Parkell C&B Metabond Quick Adhesive Cement System). Custom-made stainless steel headplates (Perelman Research Instrumentation Shop; eMachineShop) were also secured to the skull using Metabond with the remainder of the headcap being constructed with clear or black tinted Ortho-Jet dental acrylic (Lang Dental 1320CLR, 1306CLR, and 3302BLK).

### Fiber photometry recordings

Batched GCaMP fluorescence was recorded using the Neurophotometrics fiber photometry system (FP3002, MBF Bioscience), while animals performed the behavioral task. Briefly, we used time-dependent modulation in which 415- and 470-nm LEDs were alternated at a combined frequency of 80 Hz (40 Hz per wavelength) to capture isosbestic and calcium-dependent GCaMP fluorescence, respectively. Emission was collected via a 200-μm core, 0.39 NA fiber optic patch cord and detected by an internal CMOS camera with submillisecond synchronization. Excitation power at the patch cord tip was calibrated to 20 to 40 μW per wavelength using an external power meter. Recordings were acquired and initially processed using Bonsai, with behavioral events time-locked to the fluorescence signal via 5-V TTL input signals delivered from an Arduino Uno.

### Photometry analysis

Fluorescence signals were preprocessed using a custom MATLAB pipeline adapted from previously published code (*101*). Raw data were separated by channel (415-nm isosbestic and 470-nm GCaMP), and the first 400 frames were discarded to remove initial instability. Signals were then low-pass filtered at 5 Hz (fourth-order Butterworth) to reduce high-frequency noise. Slow drift due to photobleaching was corrected by fitting a cubic polynomial to each channel and computing Δ*F*/*F* as the residual divided by the fitted curve. To isolate calcium-dependent fluorescence changes, we performed a robust linear regression of the debleached isosbestic Δ*F*/*F* signal onto the debleached GCaMP Δ*F*/*F* signal.

To obtain group fluorescence traces, the Δ*F*/*F* (Δ*F*/*F*) values surrounding the event (e.g., sound onset) were averaged across trials to obtain an average fluorescence trace for each recording site. These average traces were then averaged across recording sites for each animal to obtain the an animal average fluorescence trace, which was in turn averaged across animals in a group to obtain a group fluorescence trace To quantify sound-evoked response magnitude, a robust sound-evoked peak metric was computed for each recording stream as the mean of the top 5 Δ*F*/*F* samples within the first 400 ms following sound onset minus the mean Δ*F*/*F* during the 500-ms baseline preceding sound onset.

Peak values were compared across trial types using a linear mixed-effects model with trial type as a fixed effect and a random intercept for animal [Peak ∼ TrialType + (1|Animal_ID)] to account for repeated measures within animals. Significance of the fixed effect of trial type was assessed from the fitted linear mixed-effects model using Satterthwaite’s approximation for denominator degrees of freedom. Pairwise comparisons between trial types were performed as linear contrasts on the fitted model, and resulting *P* values were corrected for multiple comparisons using the Benjamini-Hochberg false discovery rate (FDR) procedure (FDR = 0.05). The same method was used to quantify differences in evoked peaks between high- and low-frequency sounds: Comparisons were conducted using the following linear mixed-effects model: Peak ∼ SoundFrequency + (1|Animal_ID).

### Neural encoding model

To separate sensory and behavioral contributions to SPN photometry signals, we used a kernel-based linear model that included a combination of event-based and continuous predictors. Event-based predictors included trial start (aligned to light onset at trial start), sound onset, instruction (Go/NoGo, aligned to sound onset; Go coded as +0.5, NoGo coded as −0.5), hit (aligned to first lick), false alarm (aligned to first lick), and correct rejection/miss (aligned to the end of the response window). Event-based predictors were modeled as temporal kernels parameterized using six to seven raised cosine basis functions spaced uniformly across the corresponding kernel windows (see table S1). Continuous predictors included a response-withholding regressor, defined as a boxcar spanning the response window, and lick rate, computed using licks occurring within the 200 ms preceding each photometry sample. More precisely, fluorescence activity was modeled as

$$y\left(t\right)=\beta_{0}+\sum_{e\in E}\sum_{j=1}^{K_{e}}\beta_{e,j}\left(x_{e}*b_{e,j}\right)\left(t\right)+\beta_{\text{lick}}L\left(t\right)+\beta_{\text{withhold}}W\left(t\right)+\varepsilon \left(t\right)$$

where *y*(*t*) is the photometry signal (Δ*F*/*F*) at time *t*, β_0_ is the intercept, *e* indexes the event-based predictors (event set *E* = [trialStart, soundOnset, soundInstruction, Hit, FA, CRMiss]), *x_e_*(*t*) is the event time series for predictor *e*, *b*_*e*,*j*_(*t*) is the *j*th raised cosine basis function for event *e*, and (*x_e_* ∗ *b*_*e*,*j*_)(*t*) denotes convolution of the event time series with that basis function. *L*(*t*) denotes lick rate, *W*(*t*) denotes the response-withholding boxcar regressor, and ε(*t*) is the residual error. Model parameters were estimated using ridge regression, with the regularization parameter (λ) selected independently for each recording using fivefold blocked cross-validation across contiguous time segments. The base model only included the event-based predictors. The full model included all predictors.

Model performance was evaluated using cross-validated MSE on held-out data. For each recording stream, fivefold blocked cross-validation was used to compute prediction error for each model variant. To assess the contribution of additional predictors, we computed the change in cross-validated MSE (Δ*MSE*) relative to the base model. Negative Δ*MSE* values indicate improved predictive performance. Model comparisons were therefore based on out-of-sample predictive accuracy rather than parameter count, as ridge regularization complicates the application of information criteria such as Akaike information criteria (AIC) or Bayesian information criteria (BIC).

Because instruction was effect-coded (Go = +0.5, NoGo = −0.5), condition-specific sound kernels were reconstructed from the fitted model by linearly combining the sound-onset and instruction kernels. Specifically, the Go kernel was computed as $K_{\text{Go}}\left(t\right)=K_{\text{sound}}\left(t\right)+0.5K_{\text{instruction}}\left(t\right)$, and the NoGo kernel as $K_{\text{NoGo}}\left(t\right)=K_{\text{sound}}\left(t\right)-0.5K_{\text{instruction}}\left(t\right)$. These kernels were derived separately for each recording stream.

Because the mapping between instruction and sound frequency was counterbalanced across animals, frequency-specific kernels were reconstructed by remapping the instruction kernel according to each animal’s Go frequency assignment. For animals in which the Go cue corresponded to the high-frequency sound, the high kernel was defined as $K_{\text{High}}=K_{\text{sound}}+0.5K_{\text{instruction}}$ and the low kernel as $K_{\text{sound}}-0.5K_{\text{instruction}}$. For animals in which the Go cue corresponded to the low-frequency sound, this mapping was inverted.

Kernel differences were evaluated using a paired permutation max-statistic test across time points. Animal-level difference traces were sign-flipped to generate a null distribution, and the maximum absolute difference across time points was recorded for each permutation (10,000 iterations). The 95th percentile of this distribution was used to control the family-wise error rate across the kernel window.

To additionally quantify differences in kernel responses across conditions, we computed the area under the curve (AUC) of reconstructed kernels within defined time windows following sound onset. Condition-specific kernels (e.g., Go versus NoGo and High versus Low) were reconstructed for each recording stream from the fitted model as described above. For each stream, AUC was computed by integrating the kernel over the specified time window using the trapezoidal rule.

The analysis window spanned 0 to 1150 ms after sound onset and was divided into early (0 to 575 ms) and late (575 to 1150 ms) bins. For each stream, the difference in AUC between conditions (e.g., Go/NoGo) was calculated within each bin. Statistical significance was assessed using linear mixed-effects models with random intercepts for animal to account for multiple recording streams per animal. *P* values obtained from these models were corrected for multiple comparisons across bins using the Benjamini-Hochberg FDR procedure (FDR = 0.05).

To visualize the contribution of behavioral (nonkernel) regressors to the model prediction, we computed partial predictions corresponding to individual model components. For each recording stream, the fitted model coefficients for lick rate and response withholding were multiplied by their corresponding regressors to obtain predicted contributions

$${\hat{y}}_{\text{lick}}\left(t\right)=\beta_{\text{lick}}L\left(t\right),{\hat{y}}_{\text{withhold}}\left(t\right)=\beta_{\text{withhold}}W\left(t\right)$$

These traces represent the portion of the predicted fluorescence signal attributable to each regressor. Partial predictions were aligned to sound onset and averaged within stream across trials and then averaged across streams within animals and lastly across animals within each cell type. Shaded regions represent SEM across animals.

### Optogenetic manipulations

To mask effects of the stimulation light for all optogenetic experiments, we used an LED placed near the mouse’s visual field which also served as the trial start light. For bilateral optogenetic excitation with ChrimsonR, we used ∼625-nm LED light sources (Prizmatix Fiber Coupled Dual Optogenetic-LED; Orange-Red) connected to a calibrated to an output of about 5 mW as measured at the tip of the splitter patch cord (Doric Splitter Branching Patch Cord; SBP(2)_200/230/900-0.57_1m_FCM-2xMF1.25).

For optogenetic manipulations during the Go/NoGo task, we used an Arduino with custom software to deliver 250 ms of 20-Hz pulsed light time-locked to sound presentation, beginning 50 ms before sound onset and ending 50 ms after sound offset on a randomized 25% of trials. All excitatory stimulation experiments used 20-Hz pulsed light to minimize channel desensitization.

For bilateral optogenetic inhibition with ArchT, we used 532-nm laser light sources (Shanghai Dream Lasers Technology; DPSS, SDL-532-100 T) connected to a calibrated to an output of about 10 mW as measured at the tip of the splitter patch cord. For optogenetic manipulations during the Go/NoGo task, we used a similar stimulation protocol as described for excitation except we used continuous illumination. For each experiment, all animals underwent at least three sessions of opto during Go/NoGo and sessions with a *d*′ of <2 were excluded from analysis. All of the trials from these sessions were then aggregated to calculate the final behavioral metrics based on trial type.

To examine the effects of optogenetic manipulation of SPNs outside of our direct behavioral context, we ran mice in a nonoperant session within the same behavioral boxes, during which licking had no impact on water availability but was being continuously recorded. In these nonoperant control sessions, no sounds were presented throughout the duration of the session, which consisted of at least 100 stimulation trials. The box was illuminated the entire duration of the session. Throughout the session, blocks of 10 stimulation trials (in which the stimulation LED was turned on for 1 s) or 10 control trials (in which the mask light was activated) were presented. These blocks were interleaved with water blocks, during which the animal received five randomly dispensed water rewards to encourage free licking. Water delivery, stimulation, and mask light illumination occurred at a randomized interval of 1 to 10 s following trial onset.Free licking rates were calculated for each stimulation trial in the 1 s before stimulation, 1 s during stimulation, and 1 s after stimulation. These rates were then averaged to obtain the average lick rate for each animal during each respective bin.

### Muscimol inactivation

Pharmacological inhibition using muscimol was performed as previously described (*37*, *38*). Incomplete bilateral craniotomies (0.5-mm diameter) were positioned dorsally over the TS and labeled with permanent ink during initial head plating surgeries. The head plate was stereotactically positioned during this surgery and bregma remained exposed to allow for accurate injection targeting later during head fixation in our customized injection stereotax. The skull was then covered with Kwik-Sil (World Precision Instruments), and animals were singled housed to prevent exposure of the skull. Immediately before injections, animals were lightly anesthetized using isoflurane before head fixation in the stereotax. While under isoflurane, body temperature was maintained using a 30°C warming pad [Kent Scientific, Far Infrared Warming Pad with Controller (15.2-cm width × 20.3-cm length)]. The Kwik-Sil was then removed, and the craniotomies were fully opened. Injections were performed using a Nanoject II (Drummond) fitted with a pulled glass pipette, delivering 36.8 nl to each hemisphere at a rate of ∼0.6 nl/s. Either PBS vehicle or 0.5 mM muscimol (Sigma-Aldrich, M1523) was coinjected with Alexa Fluor 647–conjugated cholera toxin subunit B (0.1 mg/ml; Thermo Fisher Scientific, C34778) to enable histological verification of injection sites. Final muscimol dose per hemisphere was roughly 2 ng. Each injection was allowed to sit for 60 s to minimize any backflow before slowly removing the injection pipette. Following the final injection, the injection sites were protected with saline and Kwik-Sil, and animals were returned to their home cage which was placed on a heating pad (K&H Pet Products Small Animal Outdoor Heated Pad) to allow for 30 minutes of recovery time before behavioral testing. Following recovery, mice were run for in a nonoperant version of the behavior where licking had no impact on reward availability. In this session, animals were able to lick freely, and water was dispensed following a randomized 1-s presentation of the Go stimulus. After about 5 min (or 40 rewards), the program was switched to the actual Go/NoGo paradigm where mice were run for 30 min. On muscimol injection days, mice ran in additional washout sessions after the effects of the acute injection had largely ceased.

### Electrophysiology

Acute slice electrophysiology experiments were performed as previously described (*46*, *102*). In brief, mice were anesthetized and perfused using ACSF containing 124 mM NaCl, 2.5 mM KCl, 1.2 mM NaH_2_PO_4_, 24 mM NaHCO_3_, 5 mM Hepes, 12.5 mM glucose, 1.3 mM MgSO_4_, and 2.5 mM CaCl_2_. Following dissection, the brain was submerged in ice-cold ACSF, and coronal sections (250 μm) were made using a vibratome (Leica VT1200). Slices were then transferred to a 32°C *N*-methyl-d-glucamine (NMDG) recovery solution composed of 92 mM NMDG, 2.5 mM KCl, 1.2 mM NaH_2_PO_4_, 30 mM NaHCO3, 20 mM Hepes, 25 mM glucose, 5 mM sodium ascorbate, 2 mM thiourea, 3 mM sodium pyruvate, 10 mM MgSO_4_, and 0.5 mM CaCl_2_. Following 12- to 15-min recovery, slices were then transferred to room temperature ACSF chamber (20° to 22°C) and left for at least 1 hour before recording. During recordings, slices were fully submerged in an oxygenated (95% O_2_, 5% CO_2_) 29° to 30°C ACSF containing picrotoxin (PTX) (100 μM; Hello Bio), released at a flow rate of 1.4 to 1.6 ml/min.

For voltage-clamp recordings, recording pipettes were pulled from borosilicate glass (World Precision Instruments, TW150-3) with a tip resistance of 3 to 5 MΩ when filled with internal solution containing 115 mM CsMeSO_3_, 20 mM CsCl, 10 mM Hepes, 0.6 mM EGTA, 2.5 mM MgCl, 10 mM Na-phosphocreatine, 4 mM Na-ATP, 4 mM Na–guanosine 5′-triphosphate (GTP), 0.1 mM spermine, and 1 mM QX-314 (pH adjusted to 7.3 to 7.4 with CsOH). For current-clamp recordings, we used a K-based internal solution containing 140 mM K-gluconate, 5 mM KCl, 10 mM Hepes, 0.2 mM EGTA, 4 mM MgCl, 10 mM Na-phosphocreatine, 4 mM Na-ATP, and 0.3 mM Na-GTP (pH adjusted to 7.3 to 7.4 with KOH). To optogeneticially evoke cortical inputs, we used a full-field 470-nm illumination from a collimated LED illuminator (CoolLED, PE-300) through a 40× objective (Olympus, 0.8 NA water immersion) with a pulse width of 1 ms. Optical-evoked voltage-clamp recordings (*V*_h_ = −80 mV) were performed in the presence of PTX (100 μM), a GABA_A_ antagonist. LED intensities ranged from 0.57 to 1.65 mW/mm^2^ during the optical input output measurements.

Paired-pulse ratios (PPRs) were measured by optically evoking AMPA receptor–mediated excitatory postsynaptic currents (EPSCs). Each paired-pulse trial consisted of two light pulses of equal intensity and duration, applied at interstimulus intervals (ISIs) of 50, 100, 200, or 500 ms. All recordings were performed in the continuous presence of PTX (100 μM) to isolate glutamatergic currents. For each ISI, five trials were collected from each cell, and successive sets of trials were separated by a 10-s interval. The amplitude of the second EPSC (EPSC2) was normalized to the amplitude of the first EPSC (EPSC1) to calculate the PPR (EPSC2/EPSC1).

To test the impact of hM3D on recruitment on TS iSPNs, we injected the TS of A2a-Cre mice with Cre-dependent excitatory DREADDs (hM3Dq-mCherry) and cut 250-μm coronal TS slices after 3-week incubation. We patched iSPNs identified by their mCherry fluorescence in whole-cell current clamp configuration, injecting positive current to bring *V*_m_ = −50 mV. In the absence of synaptic blockers, we used a theta glass electrode placed within the striatal neuropil and stimulated using spike train patterns created in (*46*). We collected seven sweeps per cell (each with a unique input pattern) in ACSF, washed on 1 μM DCZ for 10 min, and then collected the same input patterns. Spiking efficiency was measured as the proportion of evoked action potentials divided by the total number of input stimuli.

### Chemogenetics methods

Nrxn1α^−/−^; A2ACre were injected in the TS with a Cre-dependent excitatory DREADDs (hM3Dq) or mCherry control virus to allow cell type–specific excitation of TS indirect pathway SPNs using the DREADDs agonist DCZ. Animals were first trained on the Go tone with sound delays of up to 2 s (see “Behavioral analysis” section) until they reached a performance criterion of *d*′ ≥ 2 for three consecutive sessions, calculated from the hit and premature rates. On the third session, mice received an intraperitoneal injection of normal saline (0.9% sodium chloride) to habituate animals to the injection procedure. The injection volume matched the volume used for DCZ administration on subsequent days. Injections were performed under brief isoflurane anesthesia 30 min before the behavioral session. Animals meeting *d*′ criteria were then advanced to Go/NoGo training.

On the first five days of Go/NoGo training, animals received an intraperitoneal injection of DCZ (100 μg/kg) under brief isoflurane anesthesia 30 min before the start of the behavioral session. DCZ dihydrochloride (HelloBio) was dissolved in normal saline (0.9% sodium chloride) to a concentration of 50 μg/ml, aliquoted into 500-μl volumes, and stored at −20°C for up to 5 weeks. On the day of use, the aliquots were wrapped in aluminium foil to protect from light and thawed at room temperature for 1 hour. The concentration of 50 μg/ml was chosen to minimize injection volume (typically 30 to 50 μl per mouse) and thereby reduce potential satiety effects during water restriction.

Behavioral performance across the five training days was quantified using false alarm rate and hit rate (described in “Behavioral analysis” section).
