# Supplementary material for: Indirect pathway neurons in the tail of the striatum regulate inhibitory control over sensory driven behavior
Source: Sci Adv. 2026 Jul 31;12(31):eaeb5352. doi: 10.1126/sciadv.aeb5352 (PMC13426436; doi:10.1126/sciadv.aeb5352)
Supplement: Supplementary file 1 — Figs. S1 to S11 Table S1 [file sciadv.aeb5352_sm.pdf]

Science Advances
AAAS

Supplementary Materials for

Indirect pathway neurons in the tail of the striatum regulate inhibitory control over sensory driven behavior

Sarah M. Ferrigno *et al.*

Corresponding author: Marc V. Fuccillo, fuccillo@pennmedicine.upenn.edu

Sci. Adv. **12**, eaeb5352 (2026)
DOI: 10.1126/sciadv.aeb5352

This PDF file includes:

Figs. S1 to S11
Table S1

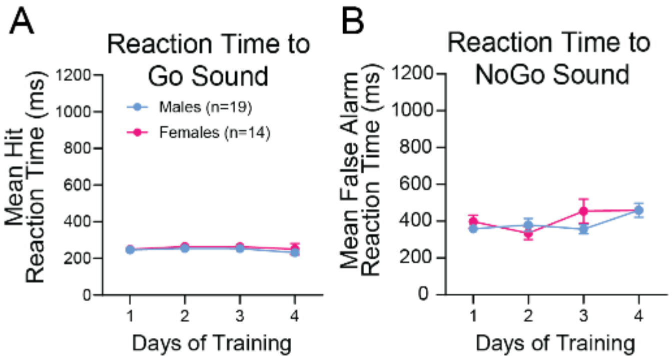

Figure S1. Stable reaction times to Go and NoGo sounds across training.

(A) Reaction times to Go sound across Go/NoGo training for male (*n* = 19) and female (*n* = 14) mice. Symbols signify group means ± SEM. Linear mixed effects model with training day and sex as fixed effects, and subject as a random effect. There were no significant main effects of either training day ($F(1.027, 23.96) = 1.74$, p = 0.1995; Geisser-Greenhouse correction applied) or sex ($F(1, 31) = 0.33$, $p = 0.57$) for hit reaction times.

(B) Reaction times to NoGo sound across Go/NoGo training for male (*n* = 19) and female (*n* = 14) mice. Symbols signify group means ± SEM. Linear mixed effects model with training day and sex as fixed effects, and subject as a random effect. There were no significant main effects of either training day ($F(2.234, 52.13)$ = 1.031, $p = 0.37$; Geisser-Greenhouse correction applied) or sex ($F(1, 31) = 0.42$, $p = 0.52$) for false alarm reaction times.

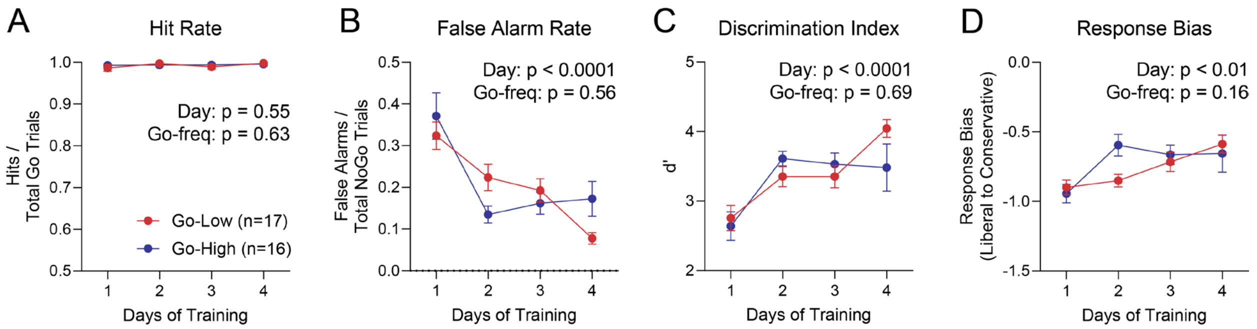

Figure S2. Behavioral performance across Go/NoGo training does not depend on Go stimulus frequency assignment.

(**A**-**D**) Behavioral metrics across the first four days of Go/NoGo training for animals trained with either the low-frequency band-limited sound as the Go cue (Go-Low; red, n = 17) or the high-frequency sound as the Go cue (Go-High; blue, n = 16). Data are shown as mean ± SEM across animals.

(**A**) Hit rate (Hits / total Go trials).

(**B**) False alarm rate (False alarms / total NoGo trials)

(**C**) Discrimination index (d')

(**D**) Response bias (criterion; more negative values indicate a more liberal response strategy).

Mixed-effects models (REML) with day of training as a repeated factor revealed a significant main effect of Day for false alarm rate, discrimination index, and response bias, but not hit rate. Go stimulus frequency assignment had no significant effect on any behavioral metric.

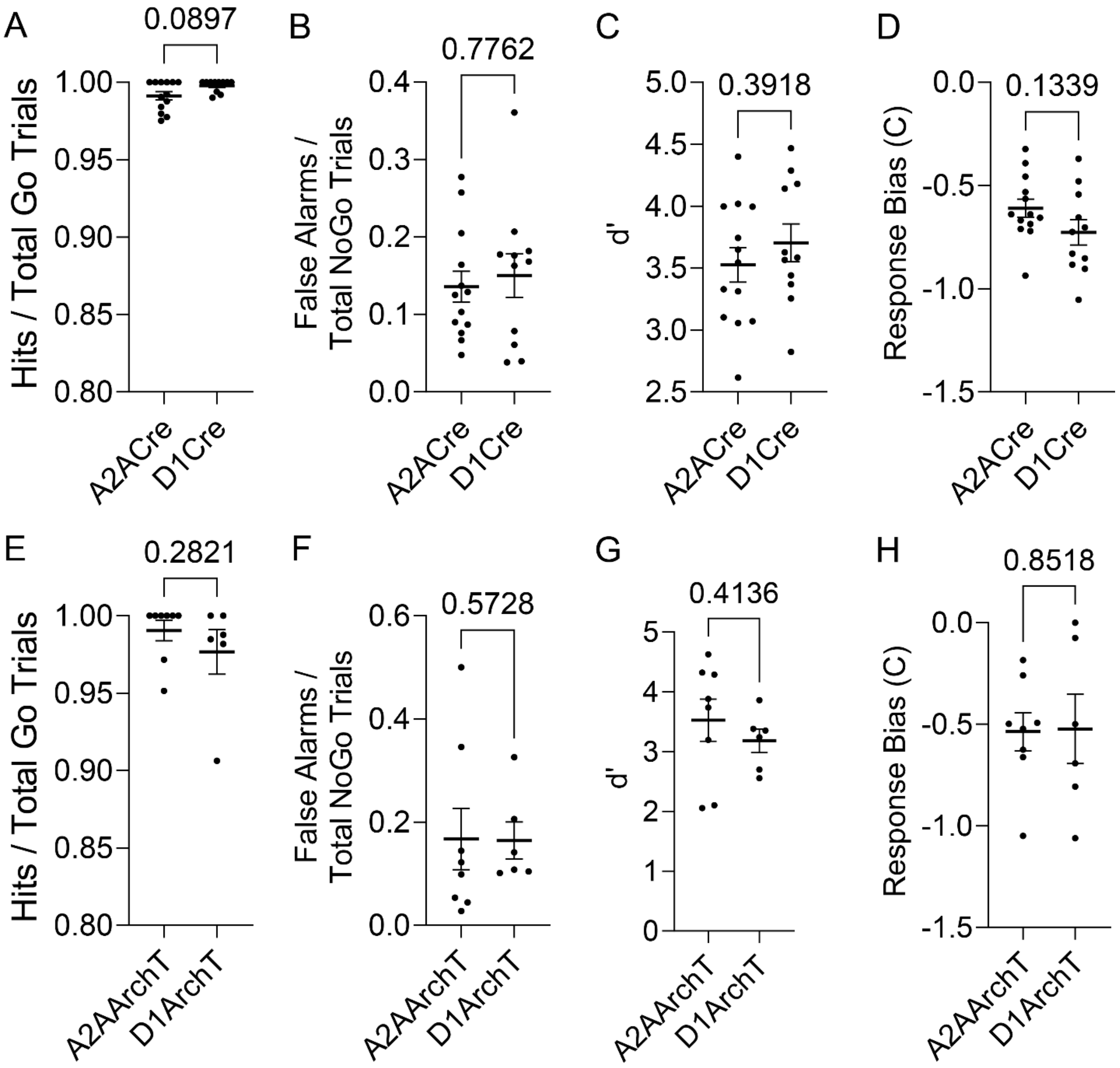

Figure S3. No genotypic differences in Go/NoGo performance across photometry and optogenetic cohorts.

(**A**-**D**) Comparison of behavioral performance between A2ACre and D1Cre mice used for photometry recordings. Metrics include hit rate (**A**), false alarm rate (**B**), discrimination index (*d′*; **C**), and response bias (c; **D**). Each point represents an individual mouse (A2ACre: *n* = 13; D1Cre: *n* = 11). Horizontal bars denote mean ± SEM.

(**E**-**H**) Comparison of behavioral performance between A2AArchT and D1ArchT mice used for optogenetic inhibition experiments. Metrics include hit rate (**E**), false alarm rate (**F**), discrimination index (*d′*; **C**), and response bias (*c*; **D**). Each point represents an

individual mouse (A2AArchT: n = 8; D1ArchT: n = 6). Horizontal bars denote mean ± SEM.

Statistical comparisons between genotypes were performed using two-tailed Mann-Whitney U tests. No significant differences were detected for any behavioral metric (p values shown in panels).

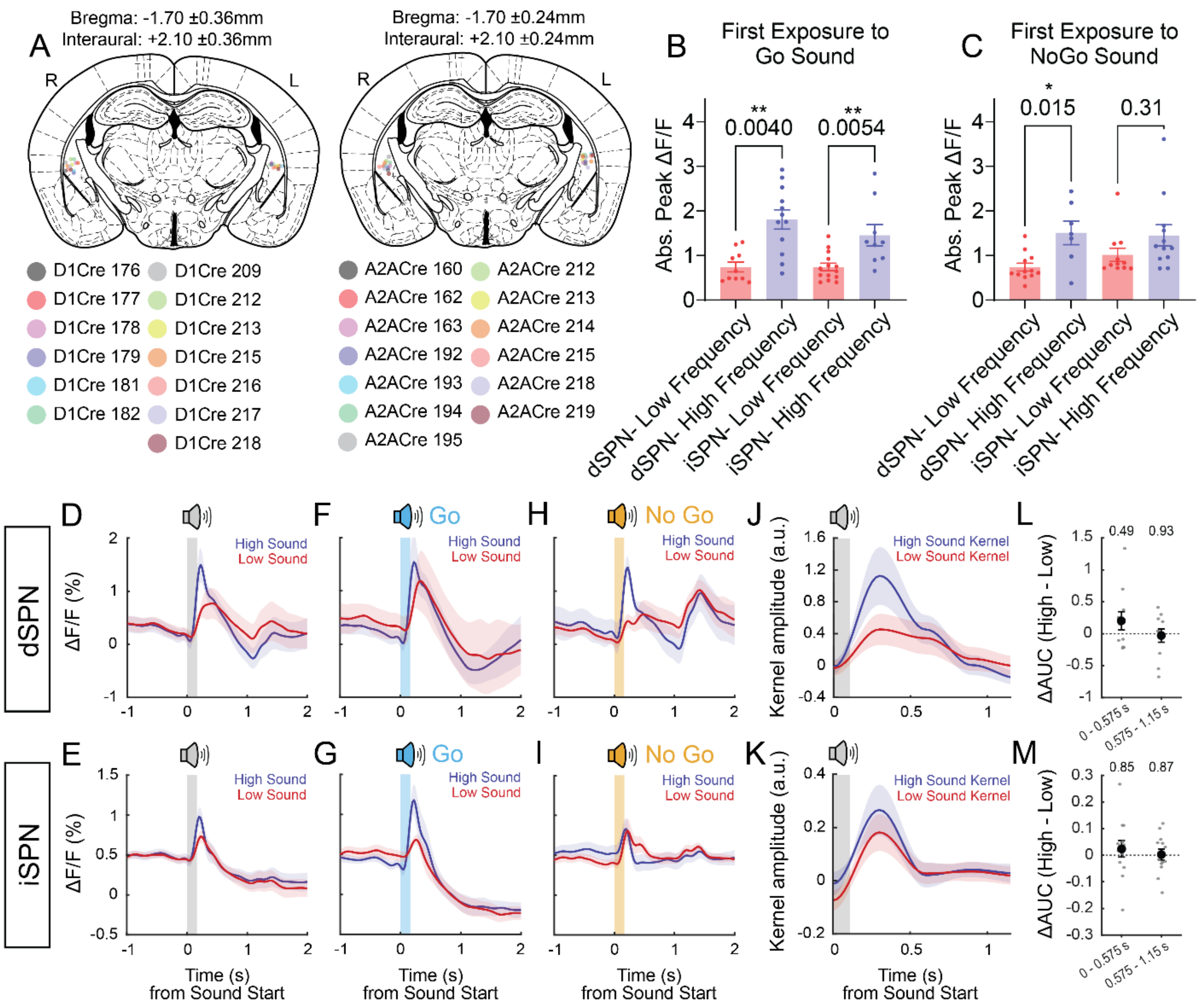

Figure S4. Photometry target region shows a modest bias toward higher sound frequencies.

(**A**) Summary fiber placements for (left) D1Cre and (right) A2ACre photometry mice. Atlas diagrams used with permission of Elsevier Science & Technology Journals, from The Mouse Brain in Stereotaxic Coordinates, Paxinos & Franklin, 2nd Edition, 2001; permission conveyed through Copyright Clearance Center, Inc.

(**B**) Mean absolute peak fluorescence values during the first day of association training for each cell-type and the assigned Go frequency. Dots signify individual recording sites [D1C: n_{low} = 6 mice/10 sites, n_{high} = 6 mice/12 sites; A2AC: n_{low} = 7 mice/14 sites, n_{high} = 5 mice/9 sites] with bars denoting group mean ± SEM. Comparisons across D1C mice were performed using a two-way mixed-effects model (REML) with Go Frequency and Hemisphere as fixed effects and Subject as a random effect; Go Frequency, p = 0.0040**; Hemisphere, p = 0.2752; interaction, p = 0.6596. Comparisons across A2AC mice, two-way mixed-effects model (REML) with Go Frequency and Hemisphere as fixed effects

and Subject as a random effect; Go Frequency, $p = 0.0054$**; Hemisphere, $p = 0.8869$; interaction, $p = 0.3099$.

(**C**) Mean absolute peak fluorescence values during correct rejection trials on the first day of Go/NoGo training for each cell-type and the assigned NoGo frequency. Dots signify individual recording sites [D1C: n_{low} = 6 mice/12 sites, n_{high} = 5 mice/7 sites; A2AC: n_{low} = 6 mice/11 sites, n_{high} = 6 mice/12 sites] with bars denoting group mean ± SEM. Comparisons across D1C mice were performed using a two-way mixed-effects model (REML) with NoGo Frequency and Hemisphere as fixed effects and Subject as a random effect; NoGo Frequency, $p = 0.0148$*; Hemisphere, $p = 0.0129$*; interaction, $p = 0.1996$. Comparisons across A2AC mice, two-way mixed-effects model (REML) with NoGo Frequency and Hemisphere as fixed effects and Subject as a random effect; NoGo Frequency, $p = 0.3106$; Hemisphere, $p = 0.0832$; interaction, $p = 0.6916$.

(**D**-**E**) Animal-level mean $\Delta F/F$ activity of dSPNs (**D**; $N = 11$ animals, 20 recording sites) and iSPNs (**E**; $N = 13$ animals, 25 recording sites) aligned to sound onset, grouped by sound frequency: High Sound (blue), Low Sound (red). Shaded regions indicate animal-level SEM. Mapping of high- and low-frequency band-limited sounds to Go and NoGo instructions was counterbalanced across animals (dSPN: Go = high in 6 animals, Go = low in 5 animals; iSPN: Go = high in 6 animals, Go = low in 7 animals). Data are from expert sessions (3 consecutive sessions $d' \geq 2$).

(**F**-**G**) Animal-level activity of dSPNs (**F**) and iSPNs (**G**) on Go trials aligned to sound onset, grouped by sound frequency.

(**H**-**I**) Animal-level activity of dSPNs (**H**) and iSPNs (**I**) on NoGo trials aligned to sound onset, grouped by sound frequency.

(**J**-**M**) Kernel-based generalized linear model (GLM) analysis separating sensory and behavioral contributions to the photometry signals (see Fig3.**G**-**H** in main paper).

(**J**-**K**) Sound-evoked kernels estimated from the Full Model for dSPNs (**J**) and iSPNs (**K**), separated by High Sound (blue) and Low Sound (red) trials. Because the mapping between instruction and sound frequency was counterbalanced across animals, frequency-specific kernels were reconstructed by remapping the instruction kernel according to each animal's Go-frequency assignment. For animals in which the Go cue corresponded to the high-frequency sound, the High kernel was defined as $K_{High} = K_{sound} + 0.5K_{instruction}$ and the Low kernel as $K_{sound} - 0.5K_{instruction}$. For animals in which the Go cue corresponded to the low-frequency sound, this mapping was inverted. Shaded regions indicate animal-level SEM. No statistically significant differences were detected using a cluster-based permutation test (see Methods).

(**L**-**M**) Within-animal differences in kernel area under the curve (ΔAUC; High – Low) computed for early (0-0.575 s) and late (0.575-1.15 s) post-sound windows for dSPNs (**L**)

and iSPNs (**M**). AUC values were compared across sound-instruction kernels using a linear mixed-effects model (*AUC* ~ *SoundFrequency* + (1|*Animal_ID*)), run separately for early and late periods. Pairwise comparisons were corrected for multiple comparisons within each cell type using the Benjamini-Hochberg false discovery rate procedure (*FDR* = 0.05). *q*-values are shown above the corresponding Δ*AUC* estimates. Points represent individual animals; black markers indicate mean ± SEM.

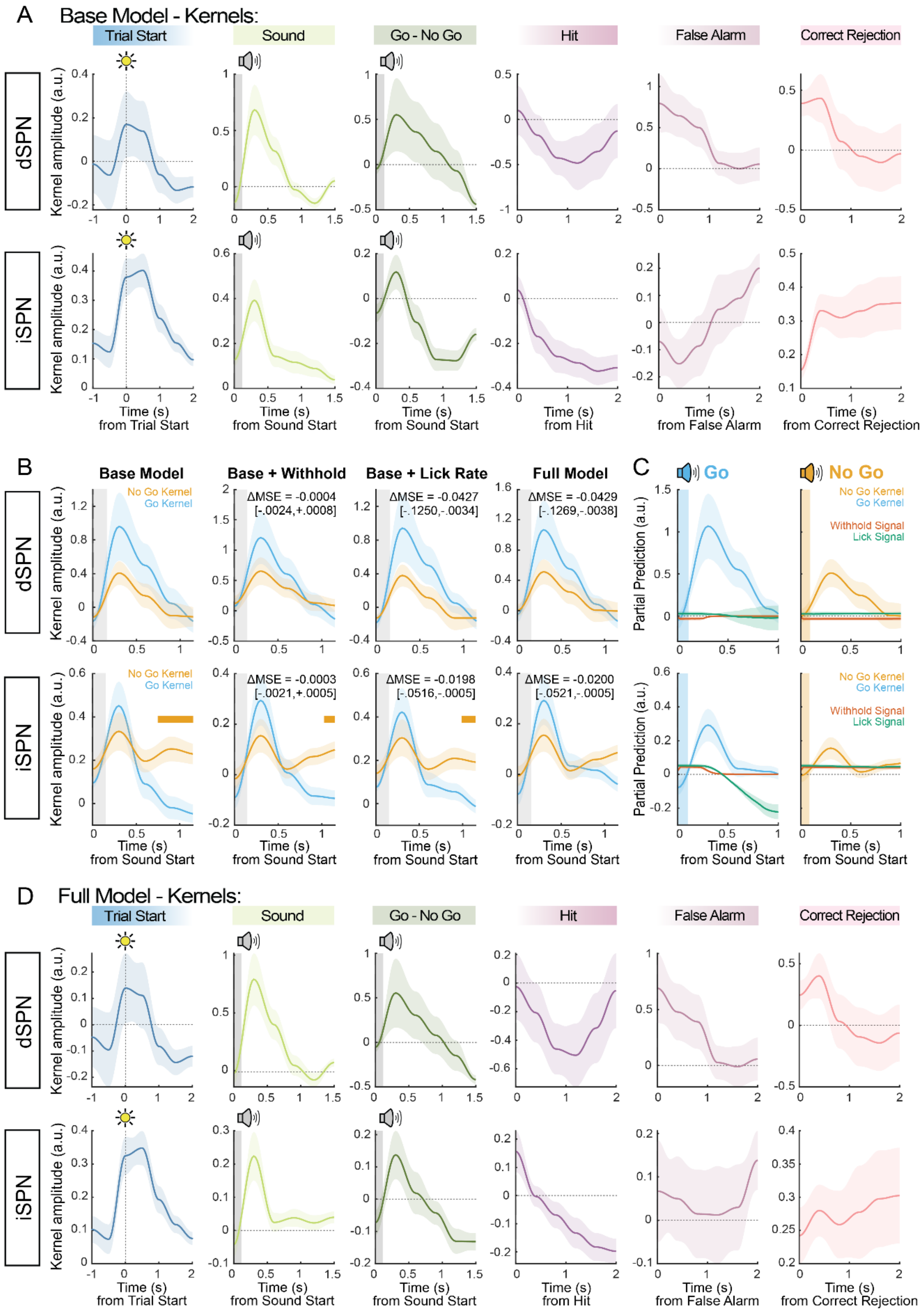

A
Base Model - Kernels:
Trial Start
Sound
Go - No Go
Hit
False Alarm
Correct Rejection
dSPN
iSPN
Kernel amplitude (a.u.)
Time (s)
from Trial Start
from Sound Start
from Hit
from False Alarm
from Correct Rejection
B
Base Model
Base + Withhold
Base + Lick Rate
Full Model
No Go Kernel
Go Kernel
ΔMSE = -0.0004
[-.0024,+.0008]
ΔMSE = -0.0427
[-.1250,-.0034]
ΔMSE = -0.0429
[-.1269,-.0038]
ΔMSE = -0.0003
[-.0021,+.0005]
ΔMSE = -0.0198
[-.0516,-.0005]
ΔMSE = -0.0200
[-.0521,-.0005]
C
Go
No Go
Partial Prediction (a.u.)
Withhold Signal
Lick Signal
D
Full Model - Kernels:

Figure S5. Addition of lick rate and response-withholding predictors improves model fit and abolishes late divergence between indirect-pathway spiny projection neuron Go and NoGo kernels.

(**A**) Kernel-based generalized linear model (GLM) analysis separating sensory and behavioral contributions to the photometry signals (see Fig3.**G**-**H** in main text) was fit independently for each photometry recording. The Base Model included event-aligned kernels for trial start, sound onset, and outcome events (Hit, False Alarm, Correct Rejection). These kernels are shown for dSPNs (top; *N* = 11 animals, 20 recording sites) and iSPNs (bottom; *N* = 13 animals, 25 recording sites). Shaded regions indicate animal-level SEM.

(**B**) NoGo and Go kernels from the Base Model (left panels), which was designed to isolate sensory contributions to the photometry signal, diverged beginning ~600 ms after sound onset, suggesting contributions from behavioral variables not included in the model. To test this possibility, we added a response-withholding boxcar regressor spanning the response window and a lick rate regressor (see Methods), both individually (middle panels), and combined (Full Model; right panels). The shaded yellow bar indicates a time period during which iSPN Go and NoGo kernels differed significantly (cluster-based permutation test; see Methods). Only inclusion of both regressors eliminated the late divergence between iSPN Go and NoGo kernels. Addition of these predictors decreased mean standard error relative to the Base Model (ΔMSE), evaluated using five-fold cross-validation on held-out data. Animal-level mean ΔMSE are shown, with minimum and maximum values indicated in brackets.

(**C**) Partial model predictions illustrating predicted contributions of individual regressors to the photometry signal at sound onset on Go (left) and NoGo (right) trials in dSPNs (top) and iSPNs (bottom). Traces show isolated contributions from lick rate (green), response withholding (orange), and Go (blue) and NoGo (yellow) sound kernels. Shaded regions indicate animal-level SEM.

(**D**) Kernels for the Full Model (Base Model + lick rate + response-withholding regressors) are shown for dSPNs (top) and iSPNs (bottom). Shaded regions indicate animal-level SEM.

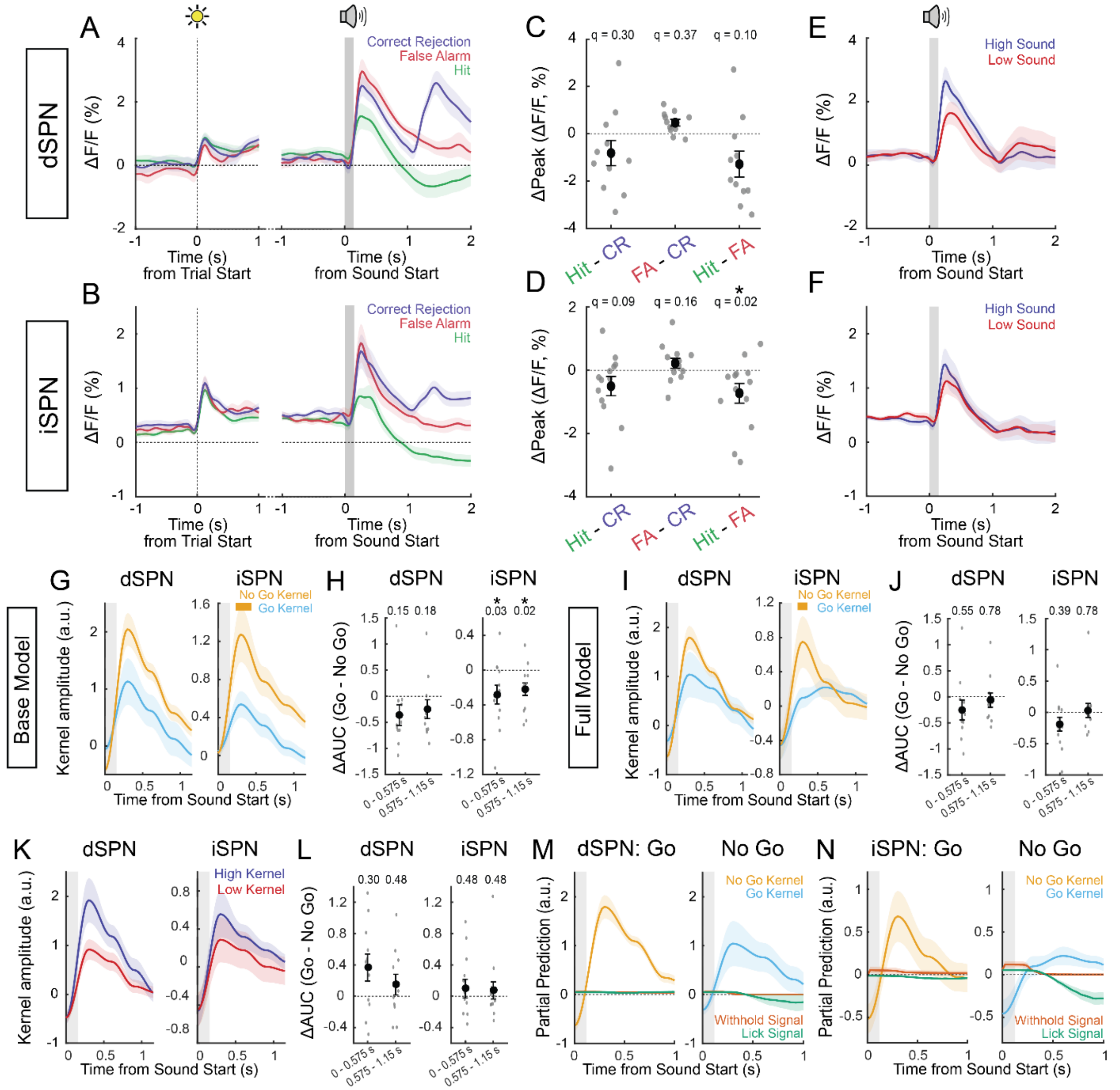

Figure S6. Early bias towards larger SPN responses to the NoGo sound on the first day of Go / NoGo training.

(**A**-**B**) Animal-level mean Δ*F*/*F* activity of dSPNs (**A**; *N* = 11 animals, 20 recording sites) and iSPNs (**B**; *N* = 13 animals, 25 recording sites) aligned to trial start (left) and sound onset (right), grouped by current trial outcome: Hit (green), False Alarm (red), and Correct Rejection (blue). Shaded regions indicate animal-level SEM. Mapping of high- and low-frequency band-limited sounds to Go and NoGo instructions was counterbalanced across animals (dSPN: Go = high in 6 animals, Go = low in 5 animals; iSPN: Go = high in 6 animals, Go = low in 7 animals). Data are from the first day of Go / NoGo training, not

expert sessions. Animals had previously been trained on the Go sound, such that this session represents their first exposure to the NoGo sound.

(**C**-**D**) Within-animal differences in evoked peak responses for dSPNs (**C**) and iSPNs (**D**). Peaks were defined per-trial as the mean of five largest Δ*F*/*F* samples within the first 400 ms following sound onset minus the mean Δ*F*/*F* during the 500 ms baseline preceding sound onset. Each gray point represents an animal; black points indicate mean ± SEM across animals. Evoked peaks were compared across trial types using a linear mixed-effects model (*Peak ~ TrialType* + (1|*Animal_ID*)). The fixed effect of trial type was significant in both dSPNs (p = 0.096, n.s.) and iSPNs (p = 0.023). Pairwise comparisons were corrected for multiple comparisons within each cell type using the Benjamini-Hochberg false discovery rate procedure (*FDR* = 0.05).

(**E**-**F**) Animal-level mean Δ*F*/*F* activity of dSPNs (**E**) and iSPNs (**F**) aligned to sound onset and grouped by sound frequency: High Sound (blue), Low Sound (red). Shaded regions indicate animal-level SEM.

(**G**-**M**) Kernel-based generalized linear model (GLM) analysis separating sensory and behavioral contributions to SPN photometry signals.

(**G**) Sound-evoked kernels estimated from the Base Model for dSPNs (left) and iSPNs (right), separated by Go (blue) and NoGo (yellow) trials. Shaded regions indicate animal-level SEM. The shaded yellow bar indicates a time period during which iSPN Go and NoGo kernels differed significantly (cluster-based permutation test; see Methods).

(**H**) Within-animal differences in kernel area under the curve (Δ*AUC*; Go – NoGo) computed for early (0-0.575 s) and late (0.575-1.15 s) post-sound windows for dSPNs (left) and iSPNs (right). AUC values were compared across sound-instruction kernels using a linear mixed-effects model (*AUC ~ SoundInstruction* + (1|*Animal_ID*)), run separately for early and late periods. Pairwise comparisons were corrected for multiple comparisons within each cell type using the Benjamini-Hochberg false discovery rate procedure (FDR = 0.05). q-values are shown above the corresponding Δ*AUC* estimates. Points represent individual animals; black markers indicate mean ± SEM.

(**I**) Sound kernels estimated from the Full Model, which additionally included behavioral regressors for lick rate and response withholding. The late Go/NoGo difference observed in iSPN kernels collapses in the Full Model, suggesting that it is better explained by lick rate and response withholding.

(**J**) Δ*AUC* (Go – NoGo) values from the Full Model computed in the same time windows as in (**H**).

(**K**) Sound kernels from the Full Model separated by sound frequency (High vs. Low). Because the mapping between instruction and sound frequency was counterbalanced

across animals, frequency-specific kernels were reconstructed by remapping the instruction kernel according to each animal's Go-frequency assignment. For animals in which the Go cue corresponded to the high-frequency sound, the High kernel was defined as $K_{High} = K_{sound} + 0.5K_{instruction}$ and the Low kernel as $K_{sound} - 0.5K_{instruction}$. For animals in which the Go cue corresponded to the low-frequency sound, this mapping was inverted. No statistically significant differences were detected using a cluster-based permutation test (see Methods).

(**L**) Within-animal Δ*AUC* values for High vs. Low sounds computed in the same early and late windows.

(**M**-**N**) Partial model predictions illustrating predicted contributions of individual regressors to the photometry signal at sound onset on Go (left) and NoGo (right) trials in dSPNs (**M**) and iSPNs (**N**). Traces show isolated contributions from lick rate (green), response withholding (orange), and Go (blue) and NoGo (yellow) sound kernels. Shaded regions indicate animal-level SEM.

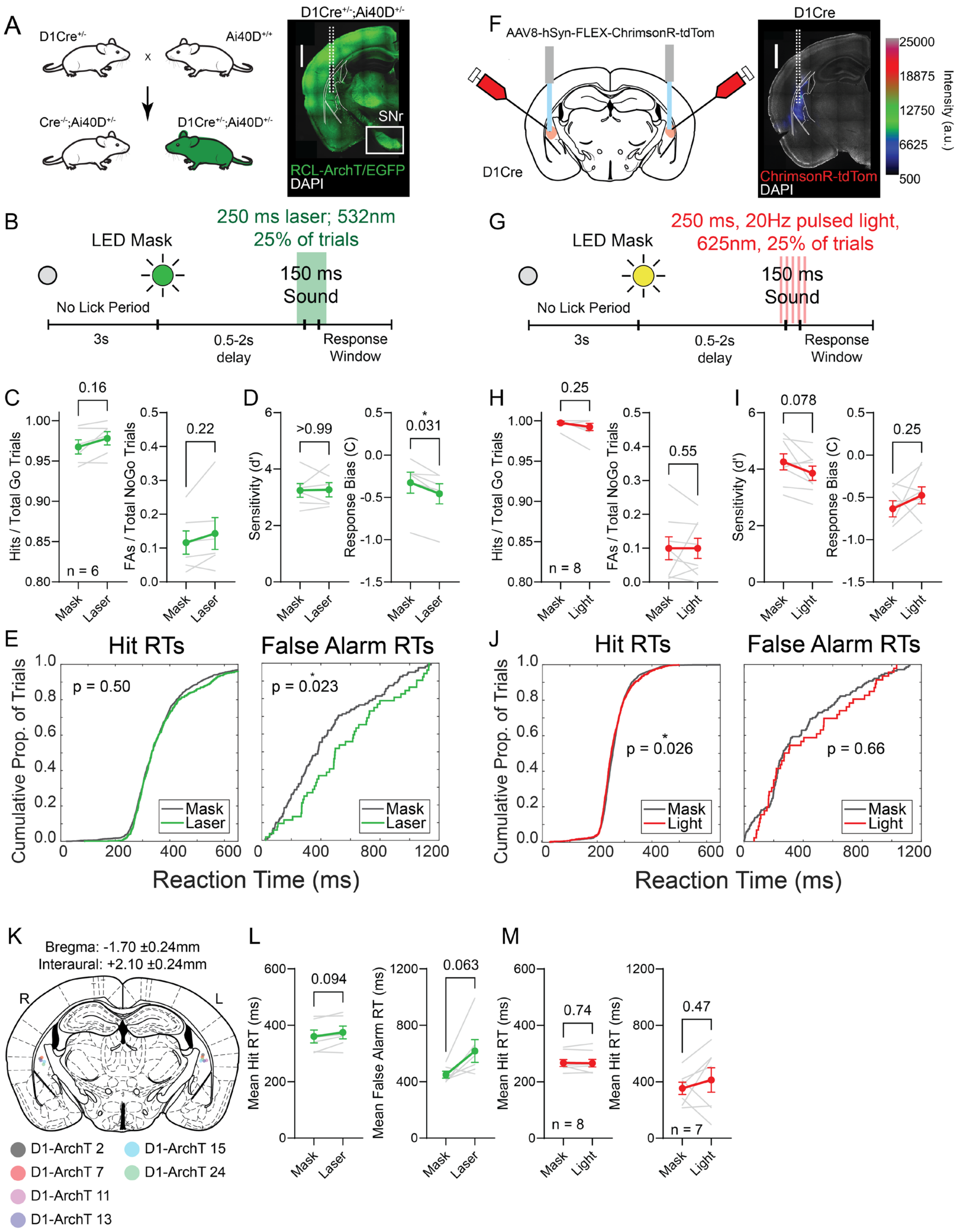

Figure S7. Manipulations of dSPNs alone have minimal impacts on behavior.

(A) Experimental breeding strategy to generate transgenic mice expressing ArchT in dSPNs with example histology image. Inset shows dSPN terminal expression in the substantia nigra pars reticulata. Scale bar is 1 mm. Illustration adapted from SciDraw under CC-BY 4.0 license (Mouse, Ethan Tyler and Lex Kravitz, DOI: 10.5281/zenodo.3925901).

(B) Schematic showing time course for laser-mediated inhibition of dSPNs during sound presentation.

(C) Hit and false alarm rates for mask control versus laser mediated inhibition trials (n = 6). Two-tailed Wilcoxon matched-pairs signed-rank test (exact): Hits (W = 15, p = 0.1562), FAs (W = 13, p = 0.2188).

(D) Discrimination sensitivity and response bias values for mask control versus laser mediated inhibition trials (n = 6). Two-tailed Wilcoxon matched-pairs signed-rank test (exact): d' (W = 1, p > 0.9999), C (W = -21, p = 0.0312*).

(E) Cumulative distributions for hit and false alarm reaction times (RT) during mask and laser inhibition conditions. Curves pool trials from multiple sessions across 6 mice [Mask: n_{hit} = 1627, n_{FA} = 119; Laser: n_{hit} = 539, n_{FA} = 52]. Differences between distributions were tested with two-sample Kolmogorov-Smirnov tests (two-sided): Hits, D = 0.0407, p = 0.5044; False Alarms, D = 0.2432, p = 0.0227*. Effect on false alarm RT was only a trend on subject-level comparison (see **L**).

(F) Experimental schematic to expressing excitatory red-shifted opsin ChrimsonR in dSPNs with example histology image. Scale bar is 1mm. Atlas images used with permission of Elsevier Science & Technology Journals, from The Mouse Brain in Stereotaxic Coordinates, Paxinos & Franklin, 2nd Edition, 2001; permission conveyed through Copyright Clearance Center, Inc.

(G) Schematic showing time course for light-mediated excitation of dSPNs during sound presentation.

(H) Hit and false alarm rates for mask control versus light mediated excitation trials (n = 8). Two-tailed Wilcoxon matched-pairs signed-rank test (exact): Hits (W = -8, p = 0.25), FAs (W = -10, p = 0.5469).

(I) Discrimination sensitivity and response bias values for mask control versus light mediated excitation trials (n = 8). Two-tailed Wilcoxon matched-pairs signed-rank test (exact): d' (W = -26, p = 0.0781), C (W = 18, p = 0.25).

(J) Cumulative distributions for hit and false alarm reaction times (RT) during mask and light conditions in excitation experiment. Curves pool trials from multiple sessions across 6 mice [Mask: n_{hit} = 2267, n_{FA} = 140; Laser: n_{hit} =768, n_{FA} = 46]. Differences between distributions were tested with two-sample Kolmogorov-Smirnov tests (two-sided): Hits, D = 0.0612, p = 0.0261*; False Alarms, D = 0.1214, p = 0.6586. Effect on hit RT was not recapitulated with subject-level comparison (see **M**).

(K) Summary fiber placements for D1-ArchT+ mice. Atlas images used with permission of Elsevier Science & Technology Journals, from The Mouse Brain in Stereotaxic Coordinates, Paxinos & Franklin, 2nd Edition, 2001; permission conveyed through Copyright Clearance Center, Inc.

(L) Average reaction time by animal for hits (left) and false alarm (right) trials across mask and laser mediated inhibition trials (n = 6). Two-tailed Wilcoxon matched-pairs signed-rank test (exact): Hits (W = 17, p = 0.0938), FAs (W = 19, p = 0.0625).

(M) Average reaction time by animal for hits (left) and false alarm (right) trials across mask and light mediated excitation trials. One animal was omitted from the false alarm comparison because they did not have any false alarms for the light on condition. Two-tailed Wilcoxon matched-pairs signed-rank test (exact): Hits (n = 8, W = 6, p = 0.7422), FAs (n = 7, W = 10, p = 0.4688).

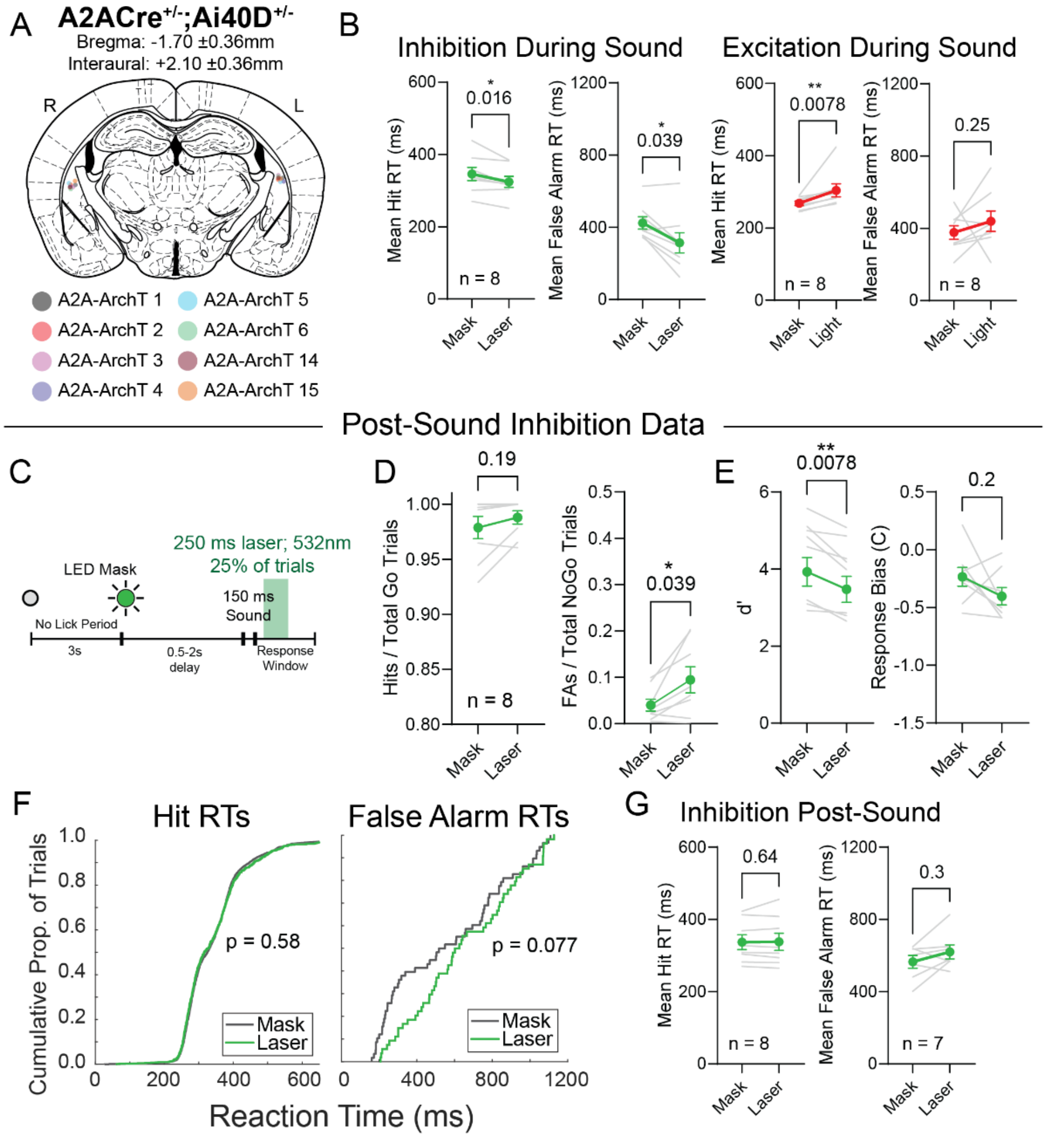

Figure S8. Post-sound inhibition of iSPNs.

- **(A)** Summary fiber placements for A2A-ArchT+ mice. Atlas images used with permission of Elsevier Science & Technology Journals, from The Mouse Brain in Stereotaxic Coordinates, Paxinos & Franklin, 2nd Edition, 2001; permission conveyed through Copyright Clearance Center, Inc.
- **(B)** (left pair) Average reaction time by animal for hits and false alarm trials across mask and laser mediated sound inhibition trials (see Fig. 4**E**; n = 8). Two-tailed Wilcoxon matched-pairs signed-rank test (exact): Hits (W = -34, p = 0.0156*), FAs (W = -30, p = 0.0391*). (right pair) Average reaction time by animal for hits and false alarm trials across mask and light-mediated excitation trials (see Fig. 4**J**; n = 8). Two-tailed Wilcoxon matched-pairs signed-rank test (exact): Hits (W = 36, p = 0.0078**), FAs (W = 18, p = 0.25).

(C) Schematic showing time course for laser-mediated inhibition of iSPNs after sound presentation.

(D) Hit and false alarm rates for mask control versus laser mediated post-sound inhibition trials (n = 8). Two-tailed Wilcoxon matched-pairs signed-rank test (exact): Hits (W = -11, p = 0.1875), FAs (W = 30, p = 0.0391*).

(E) Discrimination sensitivity and response bias values for mask control versus laser mediated post-sound inhibition trials (n = 8). Two-tailed Wilcoxon matched-pairs signed-rank test (exact): d' (W = -36, p = 0.0078**), C (W = -20, p = 0.1953).

(F) Cumulative distributions for hit and false alarm reaction times (RT) during mask and laser inhibition conditions. Curves pool trials from multiple sessions across 8 mice [Mask: n_{hit} = 2615, n_{FA} = 58; Laser: n_{hit} = 821, n_{FA} = 54]. Differences between distributions were tested with two-sample Kolmogorov-Smirnov tests (two-sided): Hits, D = 0.0309, p = 0.5844; False Alarms, D = 0.2350, p = 0.0771.

(G) Average reaction time by animal for hits (left) and false alarm (right) trials across mask and laser mediated post-sound inhibition trials. One animal was excluded from the right panel because it did not have any FA trials during the laser condition. Two-tailed Wilcoxon matched-pairs signed-rank test (exact): Hits (n = 8, W = -8, p = 0.6406), FAs (n = 7, W = 14, p = 0.2969).

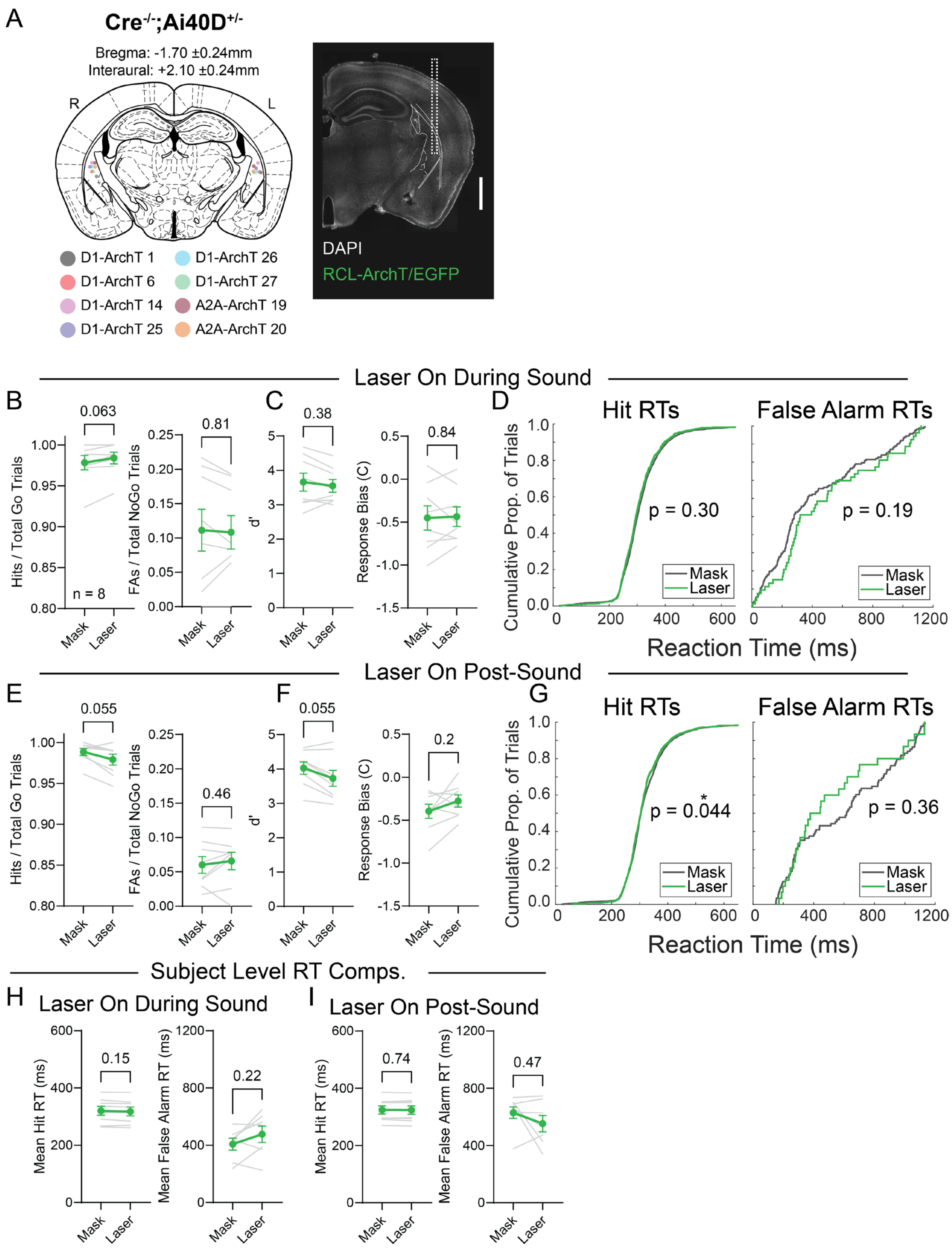

Figure S9. Cre-Negative mice do not display light-induced effects.

(A) Summary fiber placements for Cre-Negative mice along with example histological image. Scale bar is 1mm. Atlas images used with permission of Elsevier Science & Technology Journals, from The Mouse Brain in Stereotaxic Coordinates, Paxinos & Franklin, 2nd Edition, 2001; permission conveyed through Copyright Clearance Center, Inc.

(B) Hit and false alarm rates for mask control versus laser on during sound trials (n = 8). Two-tailed Wilcoxon matched-pairs signed-rank test (exact): Hits (W = 19, p = 0.0625), FAs (W = -4, p = 0.8125).

(C) Discrimination sensitivity and response bias values for mask control versus laser on during sound trials (n = 8). Two-tailed Wilcoxon matched-pairs signed-rank test (exact): *d'* (W = -14, p = 0.3828), C (W = 4, p = 0.8438).

(D) Cumulative distributions for hit and false alarm reaction times (RT) during mask and laser inhibition conditions. Curves pool trials from multiple sessions across 8 mice [Mask: n_{hit} = 2257, n_{FA} = 672; Laser: n_{hit} = 162, n_{FA} = 53]. Differences between distributions were tested with two-sample Kolmogorov-Smirnov tests (two-sided): Hits, D = 0.0427, p = 0.2955; False Alarms, D = 0.1676, p = 0.1921.

(E) Hit and false alarm rates for mask control versus laser on post-sound trials (n = 8). Two-tailed Wilcoxon matched-pairs signed-rank test (exact): Hits (W = -28, p = 0.0547), FAs (W = 12, p = 0.4609).

(F) Discrimination sensitivity and response bias values for mask control versus laser on post-sound trials (n = 8). Two-tailed Wilcoxon matched-pairs signed-rank test (exact): *d'* (W = -28, p = 0.0547), C (W = 20, p = 0.1953).

(G) Cumulative distributions for hit and false alarm reaction times (RT) during mask and laser inhibition conditions. Curves pool trials from multiple sessions across 8 mice [Mask: n_{hit} = 2356, n_{FA} = 88; Laser: n_{hit} = 767, n_{FA} = 30]. Differences between distributions were tested with two-sample Kolmogorov-Smirnov tests (two-sided): Hits, D = 0.0572, p = 0.0437; False Alarms, D = 0.1894, p = 0.3618.

(H) Average reaction time by animal for hits (left) and false alarm (right) trials across mask and laser on during sound trials. One animal was excluded from the right panel because it had no false alarms in either condition. Two-tailed Wilcoxon matched-pairs signed-rank test (exact): Hits (n = 8, W = -22, p = 0.1484), FAs (n = 7, W = 16, p = 0.2188).

(I) Average reaction time by animal for hits (left) and false alarm (right) trials across mask and laser on after sound trials. One animal was excluded from the right panel because it had no false alarms in the laser condition. Two-tailed Wilcoxon matched-pairs signed-rank test (exact): Hits (n = 8, W = -6, p = 0.7422), FAs (n = 7, W = -10, p = 0.4688).

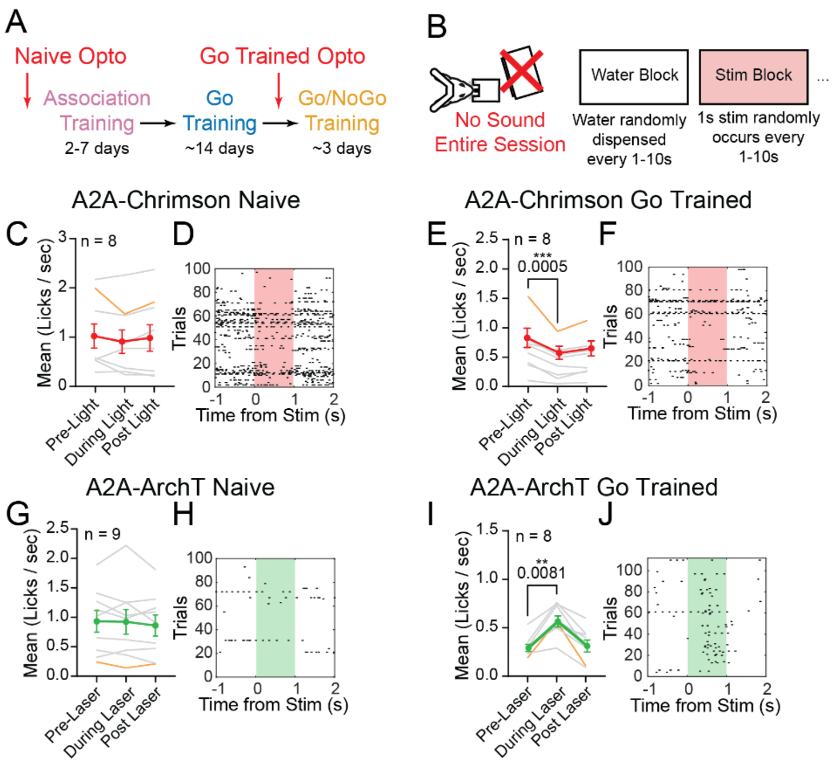

Figure S10. The indirect pathway can bias free licking probability only following training.

- **(A)** Training timeline with non-auditory opto session timepoints.
- **(B)** Schematic of the non-auditory optogenetic session. In these sessions, animals were encouraged to lick freely through random water deliveries presented in blocks alternating with stimulation periods. Illustration adapted from SciDraw under CC-BY 4.0 license (Mouse head schema, Luigi Petrucco, DOI: 10.5281/zenodo.3925903).
- **(C)** Mean lick rates before, during and after optogenetic excitation of iSPNs in naïve mice. Gray lines signify individual animals with the red symbols representing mean ± SEM. The animal highlighted in orange displayed the greatest change in lick rate during go-trained stimulation (**E**) and was used to generate the data shown in **D**. Friedman RM ANOVA on ranks (3 conditions, $n = 8$): $\chi^2(2) = 0.4516$, $p = 0.874$).
- **(D)** Example lick raster for iSPN excitation in a naïve mouse before, during and after stimulation trials. The mouse selected displayed the greatest change in mean lick rate during go-trained stimulation. Individual licks denoted by black rasters with stimulation period represented by shaded region.
- **(E)** Mean lick rates before, during and after optogenetic excitation of iSPNs in go trained mice. Gray lines signify individual animals with the red symbols representing mean ± SEM. The animal highlighted in orange displayed the greatest change in lick rate during stimulation and was used to generate the data shown in

F. Friedman RM ANOVA on ranks (3 conditions, n = 8): $\chi^2(2) = 14.25$, $p < 0.0001$****). Post hoc Dunn's (adjusted p): Pre-Light vs During-Light $p = 0.0005$***, Pre-Light vs Post-Light $p = 0.073$, During-Light vs Post-Light $p = 0.401$.

(F) Example lick raster for iSPN excitation in a go trained mouse before, during and after stimulation trials. The mouse selected displayed the greatest change in mean lick rate during go-trained stimulation. Individual licks denoted by black rasters with stimulation period represented by shaded region.

(G) Mean lick rates before, during and after optogenetic inhibition of iSPNs in naïve mice. Gray lines signify individual animals with green symbols representing mean ± SEM. The animal highlighted in orange displayed the greatest change in lick rate during go-trained stimulation (**I**) and was used to generate the data shown in **H**. Friedman RM ANOVA on ranks (3 conditions, n = 9): $\chi^2(2) = 2.667$, $p = 0.3285$).

(H) Example lick raster for iSPN inhibition in a naïve mouse before, during and after stimulation trials. The mouse selected displayed the greatest change in mean lick rate during go-trained stimulation. Individual licks denoted by black rasters with stimulation period represented by shaded region.

(I) Mean lick rates before, during and after optogenetic inhibition of iSPNs in go trained mice. Gray lines signify individual animals with green symbols representing mean ± SEM. The animal highlighted in orange displayed the greatest change in lick rate during stimulation and was used to generate the data shown in **J**. Friedman RM ANOVA on ranks (3 conditions, n = 8): $\chi^2(2) = 12$, $p = 0.0011$**). Post hoc Dunn's (adjusted p): Pre-Laser vs During-Laser $p = 0.0081$**, Pre-Laser vs Post-Laser $p > 0.9999$, During- Laser vs Post-Laser $p = 0.0081$**.

(J) Example lick raster for iSPN inhibition in a go trained mouse before, during and after stimulation trials. The mouse selected displayed the greatest change in mean lick rate during go-trained stimulation. Individual licks denoted by black rasters with stimulation period represented by shaded region.

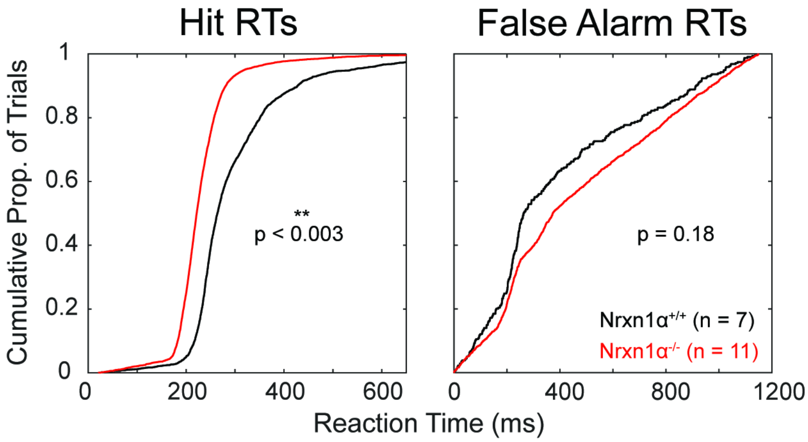

Figure S11. Reaction times on Hit and False Alarm trials in Nrxn1α$^{+/+}$ and Nrxn1α$^{-/-}$ animals during Go/NoGo training. Cumulative distribution plots showing reaction times on all Hit (left; Nrxn1α$^{+/+}$ n_{hit} = 3463, Nrxn1α$^{-/-}$ n_{hit} = 5554) and False Alarm (right; Nrxn1α+/+ n_{FA} = 314, Nrxn1α-/- n_{FA} = 1645) trials across Nrxn1α$^{+/+}$ (n = 7; black) and Nrxn1α$^{-/-}$ (n = 11; red) animals throughout NoGo training. Hit reaction times per animal were significantly shorter in Nrxn1α$^{-/-}$ mice than in Nrxn1α$^{+/+}$ mice (Wilcoxon rank-sum test, W = 98, p < 0.003). False Alarm reaction times per animal did not differ across genotypes (Wilcoxon rank-sum test, W = 51, p = 0.18).

Supplemental Tables:

Predictor	Window size PRE (s)	Window size POST (s)	Number of splines
**Trial start** (light onset)	-1	2	7
**Sound onset**	0	1.5	6
**Go/NoGo instruction** (aligned to Sound onset)	0	1.5	6
**Hit** (first lick)	0	2	6
**False Alarm** (first lick)	0	2	6
**Correct rejection** (end of response window)	0	2	6
**Response withholding**	(continuous regressor)		
**Lick rate**	(continuous regressor)		

Table S1. Details on model predictors. Window size PRE/POST indicates the extent of the kernel window before and after the event to which the predictor is aligned. “Number of splines” denotes the number of raised cosine basis functions used to parameterize each kernel. Knots locations were spaced uniformly across the kernel window.
